# Proximity-Labeling Reveals Novel Host and Parasite Proteins at the *Toxoplasma* Parasitophorous Vacuole Membrane

**DOI:** 10.1128/mBio.00260-21

**Published:** 2021-11-09

**Authors:** Alicja M. Cygan, Pierre M. Jean Beltran, Alma G. Mendoza, Tess C. Branon, Alice Y. Ting, Steven A. Carr, John C. Boothroyd

**Affiliations:** a Department of Microbiology and Immunology, Stanford School of Medicine, Stanford California, USA; b Broad Institute of MIT and Harvard, Cambridge, Massachusetts, USA; c Department of Chemistry, Stanford University, Stanford, California, USA; University at Buffalo; Duke University School of Medicine

**Keywords:** *Toxoplasma*, proximity-labeling, quantitative proteomics

## Abstract

Toxoplasma gondii is a ubiquitous, intracellular parasite that envelops its parasitophorous vacuole with a protein-laden membrane (PVM). The PVM is critical for interactions with the infected host cell, such as nutrient transport and immune defense. Only a few parasite and host proteins have so far been identified on the host-cytosolic side of the *Toxoplasma* PVM. We report here the use of human foreskin fibroblasts expressing the proximity-labeling enzyme miniTurbo, fused to a domain that targets it to this face of the PVM, in combination with quantitative proteomics to specifically identify proteins present at this interface. Out of numerous human and parasite proteins with candidate PVM localization, we validate three parasite proteins (TGGT1_269950 [GRA61], TGGT1_215360 [GRA62], and TGGT1_217530 [GRA63]) and four new host proteins (PDCD6IP/ALIX, PDCD6, CC2D1A, and MOSPD2) as localized to the PVM in infected human cells through immunofluorescence microscopy. These results significantly expand our knowledge of proteins present at the *Toxoplasma* PVM and, given that three of the validated host proteins are components of the ESCRT (endosomal sorting complexes required for transport) machinery, they further suggest that novel biology is operating at this crucial host-pathogen interface.

## INTRODUCTION

Toxoplasma gondii, the prototypical model organism for the phylum Apicomplexa, is uniquely capable of infecting virtually any nucleated cell in almost any warm-blooded animal. This ubiquitous intracellular parasite can be especially dangerous for immunocompromised individuals and the developing fetus and is also a significant veterinary pathogen (1). In addition to its medical importance, *Toxoplasma* is a powerful model organism for studying the biology of intracellular parasitism, due to its genetic tractability and ease of culture.

As an obligate intracellular organism, *Toxoplasma* relies on host cell resources in order to replicate. Like other major pathogenic species of the phylum Apicomplexa (e.g., *Cryptosporidium* spp., *Plasmodium* spp.), *Toxoplasma* has evolved to extensively modify its intracellular environment in order to establish a replicative niche, the parasitophorous vacuole (PV), in its host. The PV is necessary to facilitate *Toxoplasma*’s intracellular development and for the avoidance of host defenses. Among other pathogenic processes, *Toxoplasma* tachyzoites, the rapidly dividing forms of the parasite responsible for acute infection, remodel the host cytoskeleton (e.g., vimentin and microtubule organizing center [2–4]), recruit host organelles (e.g., mitochondria and endoplasmic reticulum [5, 6]), scavenge essential nutrients and host resources (e.g., tryptophan, cholesterol, and iron [7–9]), actively manipulate the host transcriptome to support increased metabolism and inhibit the innate immune response (e.g., accumulation of the host “master regulator” c-Myc [10, 11]), and even physically inactivate host protein defenses mounted at the PV (e.g., inactivation of interferon gamma [IFN-γ]-induced immunity-related GTPases in murine cells [12, 13]).

Much of this host cell manipulation relies on parasite effector proteins (e.g., ROP18, GRA15, GRA17/23, MAF1) introduced into the host cell during infection (recently reviewed in reference 14) and located at the parasitophorous vacuole membrane (PVM), the interface between the developing parasites and host cells. Exposed to the host cytosol, these parasite effectors can interact with various host processes (recently reviewed in reference 15). Despite the critical importance of the parasite-host interactions occurring at the PVM, only a small number of proteins at this boundary have thus far been identified. This lack of knowledge has hampered our ability to mechanistically study interesting and potentially disease-relevant aspects of parasite biology, including processes that might be amenable to parasite- and host-targeted drugs.

To address this lack, we have utilized advances in spatial proteomics to identify host and parasite proteins specifically present at the host cytosolic side of the PVM. Proximity-labeling-based methods have successfully been used to identify proteins within various *Toxoplasma* cellular compartments, including the PV lumen (16, 17), relying on expression of the crucial biotin-ligase enzymes fused to parasite-expressed proteins. To date, however, no study has used this approach to identify novel proteins specifically present on the host-cytosolic face of the PVM. By fusing the promiscuous biotin-labeling protein, miniTurbo (18), to a domain that targets it to the PVM and engineering host cells to express this fusion, we have been able to generate high-confidence lists of candidate host and parasite proteins present at the host-cytosolic face of the PVM. From a subset of this candidate pool, we have validated three parasite and four host proteins as so localized, including several that indicate that previously undescribed processes are occurring at this all-important interface.

## RESULTS

### Validation of the miniTurbo system in infected HFFs.

**FIG 1:**
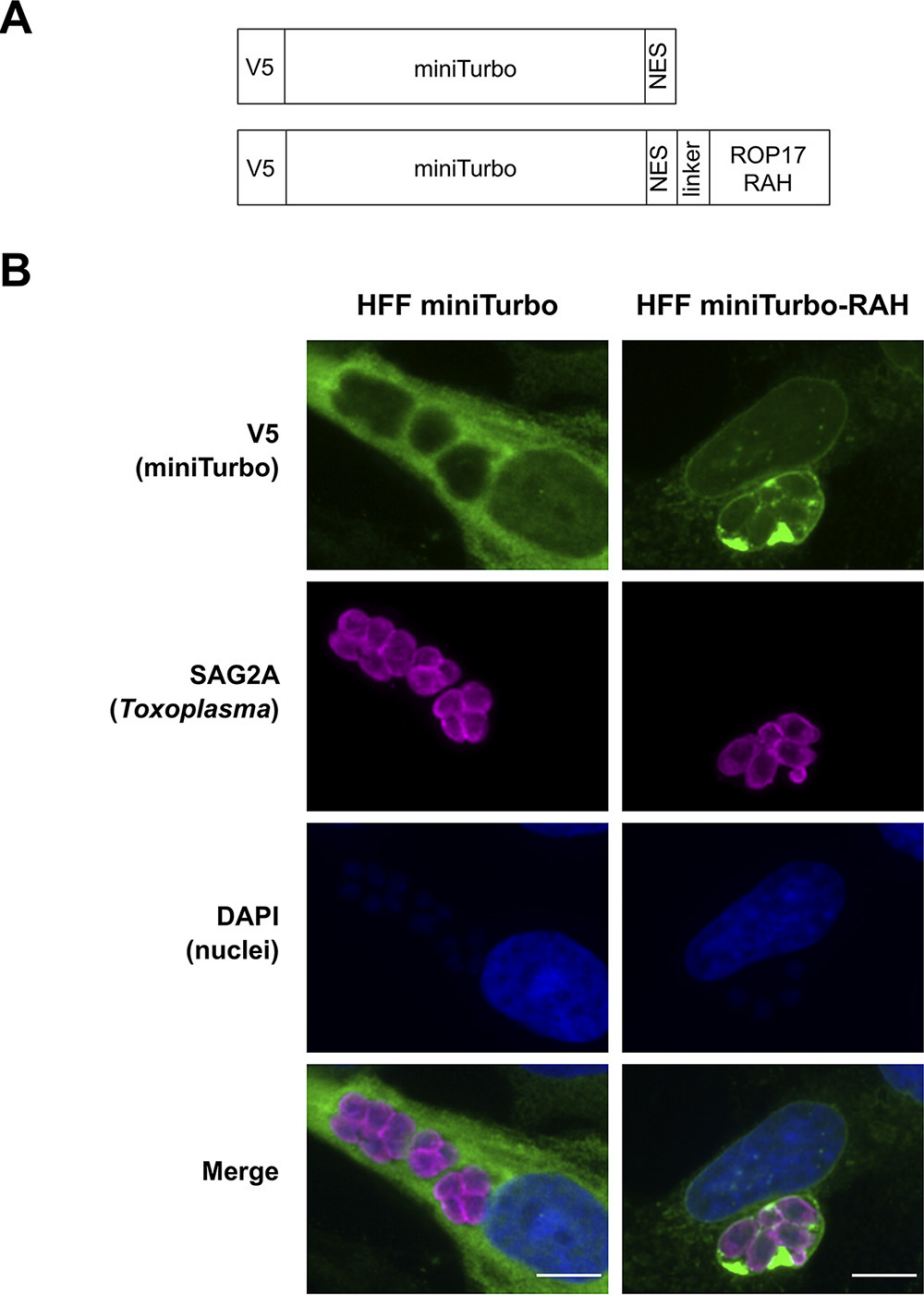
The arginine-rich-amphipathic helix domain (RAH) of ROP17 is sufficient to localize miniTurbo to the *Toxoplasma* PVM in infected cells. (A) Schematic of the miniTurbo fusion proteins expressed in HFFs depicting the epitope tag (V5), miniTurbo ligase (miniTurbo), nuclear export signal (NES), flexible linker (linker [amino acids KGSGSTSGSG]), and RAH domain of ROP17 (ROP17 RAH, [amino acids 104 to 223]). (B) Representative immunofluorescence microscopy images of tachyzoite-infected HFF populations stably expressing miniTurbo fusion proteins depicted in panel A. RHΔ*hpt*Δ*ku80* tachyzoites were allowed to infect the corresponding HFFs for 24 h before the infected monolayers were fixed with methanol. The miniTurbo fusion proteins were detected with mouse anti-V5 antibodies (green), tachyzoites were detected with rabbit anti-SAG2A antibodies (magenta), and host and parasite nuclei were visualized using DAPI (blue). Scale bar is 10 μm.

To identify host and parasite proteins exposed to the host cytosol at the PVM, we utilized enzyme-catalyzed proximity-labeling via the promiscuous biotin ligase, miniTurbo, a recently developed variant of BioID (18, 19). To target miniTurbo to the PVM, we fused V5-tagged miniTurbo-nuclear export signal (NES) to the arginine-rich-amphipathic helix (RAH) domain (amino acids 104 to 223) of the *Toxoplasma* ROP17 protein (Fig. 1A), which had previously been shown to be capable of localizing mCherry to the PVM when heterologously expressed in human cells (20). The NES was included to facilitate the cytoplasmic localization needed for miniTurbo in these experiments. We stably introduced this construct, as well as V5-tagged miniTurbo-NES without the RAH domain as a control, into human foreskin fibroblasts (HFFs) using lentiviral transduction to generate cell lines stably expressing one or the other of the miniTurbo proteins (here referred to as “HFF miniTurbo-RAH” and “HFF miniTurbo,” respectively).

We assessed the localization of miniTurbo in these cells infected with *Toxoplasma* tachyzoites (RH strain) using an immunofluorescence assay (IFA). The results (Fig. 1B) show a diffuse, cytosolic staining pattern in HFF miniTurbo cells, consistent with the localization of this construct in other human cell lines (18), with no apparent association with parasite vacuoles. In HFF miniTurbo-RAH cells, however, miniTurbo staining was highly concentrated at the PVM, being seen even “between” parasites, likely within *Toxoplasma*’s nanotubular intravacuolar network (IVN), the lumens of which are thought to be at least partially contiguous with the host cytosol (20, 21). Thus, the ROP17-RAH domain appears sufficient to target miniTurbo to the PVM in infected cells. In addition, miniTurbo-RAH staining was seen around the nuclear envelope, consistent with the localization previously observed for fluorescent protein fusions with the RAH domains of related *Toxoplasma* proteins (20). This attraction to the host nuclear envelope may be due to the nature in which *Toxoplasma* RAH domains of ROP2 family proteins (i.e., ROP2, ROP5, ROP17, ROP18, etc.) achieve their specificity for the PVM, the exact mechanism of which is unknown but hypothesized to be due to an attraction of the amphipathic helices to the strong negative curvature of the PVM (as a consequence of the IVN) and to a lesser extent attracted to the membranous invaginations of the host nucleus (22). It is notable, however, that the RAH domain is still sufficient to bring fluorescent protein fusions to the PVM in the absence of a mature IVN, leaving open the possibility that a parasite-specific protein or lipid on the PVM may at least partially be driving this association (20).

**FIG 2:**
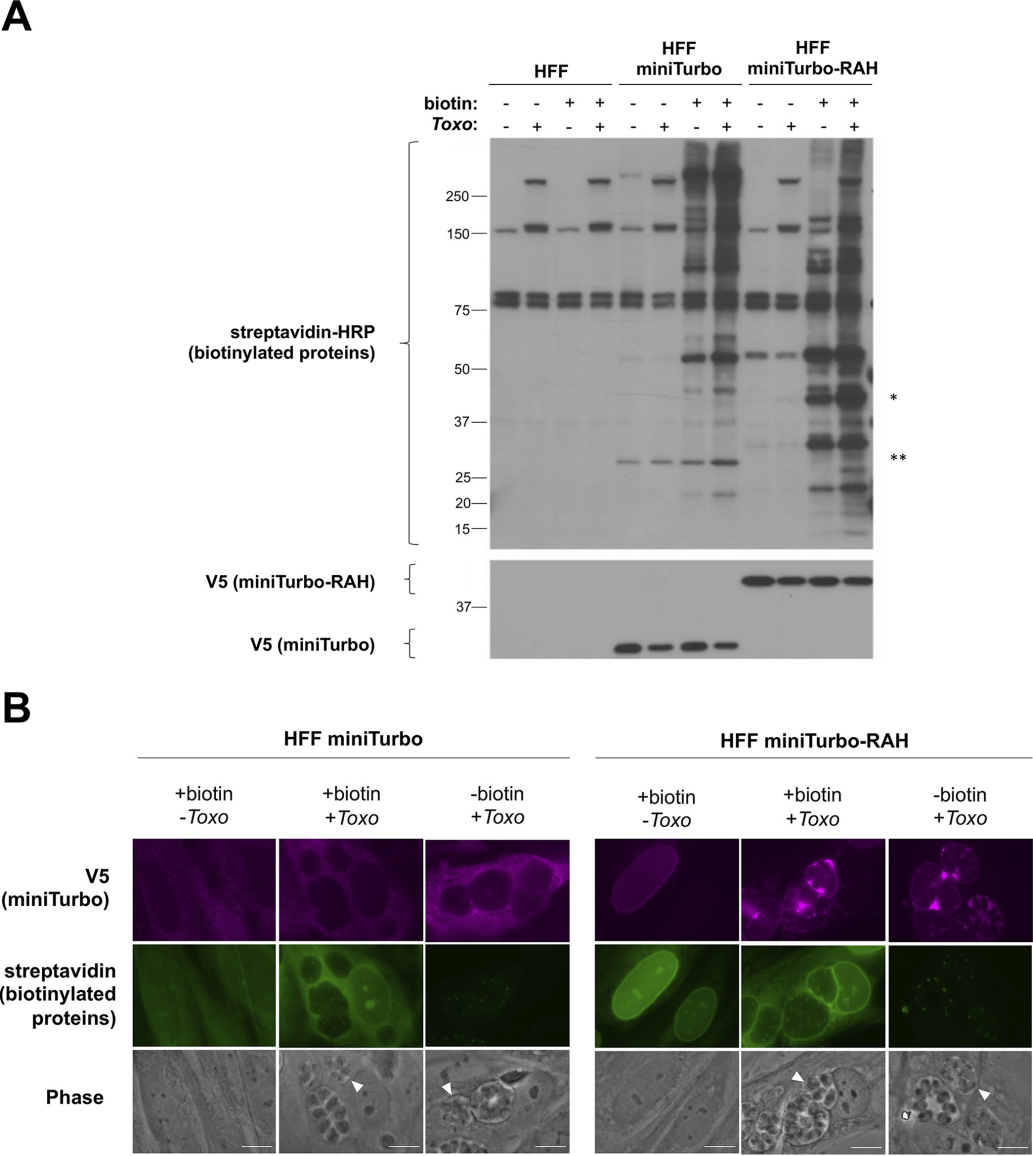
miniTurbo can biotinylate proteins in human foreskin fibroblasts. (A) Western blot of whole-cell lysates. The indicated lines of HFFs were either infected or left uninfected (+/–) with RHΔ*hpt*Δ*ku80* tachyzoites for 24 h and then incubated with or without (+/–) 50 μg of biotin for 1 h. Biotin labeling was terminated by cooling the cells to 4°C and washing away excess biotin. Whole-cell lysates (2 μg) were subsequently prepared and blotted with streptavidin-HRP to visualize biotinylated proteins and mouse anti-V5 antibodies to visualize expression of the miniTurbo fusion proteins. Bands present in the non-miniTurbo-expressing HFFs and the HFFs not incubated with biotin represent endogenously biotinylated host and parasite proteins. * denotes the location on the streptavidin blot at the approximate size of miniTurbo-RAH, and ** denotes the location on the streptavidin blot at the approximate size of miniTurbo. Approximate migration of a ladder of size standards (sizes in kDa) is indicated. (B) Representative immunofluorescence microscopy images of HFF populations stably expressing miniTurbo fusion proteins. The indicated line of HFFs was either infected with RHΔ*hpt*Δ*ku80* tachyzoites or left uninfected for 24 h and then subjected to a 1-h incubation with biotin as described in panel A. After washing, the monolayers were fixed with methanol. The miniTurbo fusion proteins were detected with mouse anti-V5 antibodies (magenta), biotinylated proteins were detected with streptavidin-HRP (green), and the entire monolayer was visualized with phase microscopy. White arrowheads indicate a representative PV. Scale bar is 10 μm.

To test the functionality of the miniTurbo and miniTurbo-RAH fusions in the host cytosol and at the PVM, we infected HFF, HFF miniTurbo, and HFF miniTurbo-RAH cells with tachyzoites, initiated labeling with the addition of exogenous biotin for 1 h, and then assessed biotinylation by Western blot analysis and IFA. Streptavidin blot analysis of whole-cell lysates (Fig. 2A) shows that in HFFs that lack miniTurbo, only a few host and parasite proteins are readily observed, presumably endogenously biotinylated since their detection is not dependent on the addition of exogenous biotin. In contrast, substantial biotin labeling was observed in both miniTurbo and miniTurbo-RAH cells, and this additional signal was dependent on the presence of biotin. Staining for the V5 tag confirmed the presence of miniTurbo of the expected mass in these cells (Fig. 2A).

To confirm the biotinylating activity in these cells, fluorescent imaging of biotinylated proteins was performed (Fig. 2B). This, too, revealed clearly detectable biotinylation in miniTurbo- and miniTurbo-RAH-expressing cells after 1 h of biotin-labeling that was dependent on the presence of exogenous biotin and largely colocalized with the miniTurbo fusion proteins. In untreated cells, we only observed punctate staining within the parasites, consistent with previous observations of endogenously biotinylated proteins present in the *Toxoplasma* apicoplast (23). Notably, less streptavidin staining was observed between parasites where the miniTurbo signal (anti-V5) was strongest in miniTurbo-RAH cells. We speculate that this may be due to a unique redox, pH, or endogenous nucleophile (free amine/lysine availability) environment within the IVN that may influence the activity of miniTurbo, as has been described in other cellular compartments (18). Alternatively, the IVN may be relatively inaccessible to the biotin, which might be preferentially utilized by the miniTurbo-RAH present where the IVN nanotubes open into the host cytosol, or this may simply be an artifact due to poor accessibility of streptavidin in fixed cells. Regardless, streptavidin signal was still strongly observed at the PVM in miniTurbo-RAH cells, making the system well suited to our goal of exploring this interface.

**FIG 3:**
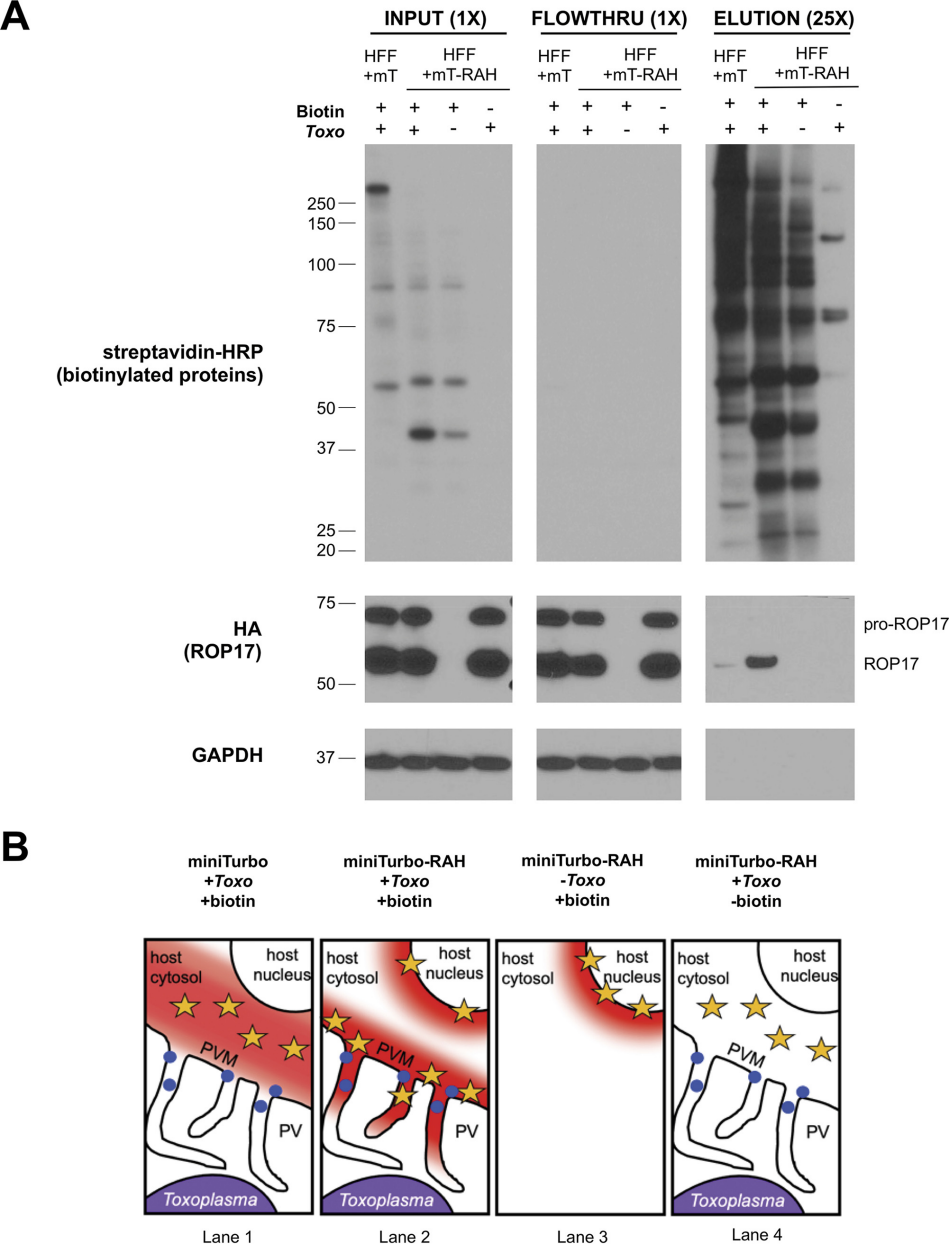
The *Toxoplasma* PVM-localized protein ROP17 is enriched in miniTurbo-RAH cells. (A) Western blot of whole-cell lysates and subsequent elutions after streptavidin affinity purification. The indicated line of HFFs was either infected or left uninfected (+/–) with RH*Δrop17*::ROP17-3xHA tachyzoites for 24 h and then incubated with or without (+/–) 50 μg of biotin for 1 h. Whole-cell lysates were subsequently prepared, and 200 μg of each lysate was incubated with streptavidin-coated magnetic beads overnight. The beads were washed and proteins were eluted from the beads by boiling in the presence of 2 mM biotin and BME. Samples of the input lysate, unbound lysate (flowthrough), and eluates were separated on an SDS-PAGE gel and blotted with streptavidin-HRP to visualize all biotinylated proteins, mouse anti-HA-HRP antibody to visualize ROP17-3xHA, and mouse anti-GAPDH to visualize host protein as a loading control. 25× indicates that the lanes containing eluted material had 25-fold more material loaded compared to input and flowthrough lanes (1×). Approximate migration of a ladder of size standards (sizes in kDa) is indicated. (B) Cartoon depiction of expected regions of biotinylation (depicted by red cloud) near the *Toxoplasma* PVM and their proximity to the localization of ROP17 (blue dots) at the PVM based on miniTurbo fusion localization (yellow stars) and addition of biotin.

We next determined the labeling specificity of known PVM proteins by miniTurbo-RAH. We infected HFF miniTurbo-RAH and HFF miniTurbo cells with RHΔ*rop17*::ROP17-3xHA tachyzoites, followed by biotin labeling and enrichment of biotinylated proteins using streptavidin-conjugated beads. Streptavidin blot analysis of the input whole-cell lysate, flowthrough from the purification, and eluted proteins showed a strong enrichment for ROP17 within the infected, treated miniTurbo-RAH eluate only, as expected given its known PVM localization (Fig. 3A). A much smaller amount of ROP17 was detected in the miniTurbo eluate, consistent with cytosolic miniTurbo having access to ROP17, but to a lesser extent than when concentrated at the PVM (cartoon depiction in Fig. 3B). Note that ROP17 exists as both an immature proprotein and a mature, proteolytically processed protein that is generated within the parasites’ nascent rhoptries prior to release (24). It is as expected, therefore, that only the mature form was detected in the miniTurbo-RAH eluate, although we did not examine this point exhaustively and so cannot exclude the possibility that minute amounts of the proprotein might be present.

To confirm and extend these results, we next asked whether another PVM-localized parasite protein, MAF1b, is also enriched in the infected miniTurbo-RAH eluate over the miniTurbo eluate. The results (Fig. S1) showed this prediction was borne out. Furthermore, a silver stain of the total protein material from the eluates revealed minimal protein background in the miniTurbo-minus and biotin-minus negative controls, indicating that our wash conditions were sufficiently stringent to remove most nonspecific background binders (Fig. S1). All combined, these results indicate that ROP17-3xHA and endogenous MAF1b, and likely other PVM-associated proteins, are preferentially biotinylated by HFF miniTurbo-RAH versus HFF miniTurbo cells and that we could thus use our streptavidin enrichment pipeline followed by quantitative ratiometric analysis to compare protein abundances between the conditions to infer PVM localization.

10.1128/mBio.00260-21.3 FIG S1 The *Toxoplasma* PVM-localized protein MAF1b is enriched in miniTurbo-RAH cells. (INPUT) The indicated line of HFFs was either infected or left uninfected (+/–) with RHΔ*ku80*Δ*hpt* tachyzoites for 24 h and then incubated with or without (+/–) 50 μg of biotin for 1 h. Whole-cell lysates were subsequently prepared, and a sample was run on SDS-PAGE gel and blotted with streptavidin-HRP to visualize all biotinylated proteins and mouse anti-V5 antibodies to visualize miniTurbo expression. (ELUTION) Silver stain and Western blots of eluates after streptavidin affinity purification. First, 100 μg of each input lysate was incubated with streptavidin-coated magnetic beads overnight. The beads were washed, and proteins were eluted from the beads by boiling in the presence of 2 mM biotin and BME. Half of each elution was run on an SDS-PAGE gel and blotted with rabbit anti-MAF1b antibodies and mouse anti-GAPDH antibodies to visualize enrichment of these proteins. The remaining halves of each elution were run on a separate SDS-PAGE gel and stained with silver to visualize total protein material eluted from the beads. Approximate migration of a ladder of size standards (kDa) is indicated. Download FIG S1, PDF file, 0.8 MB. Copyright © 2021 Cygan et al. 2021 Cygan et al. https://creativecommons.org/licenses/by/4.0/ This content is distributed under the terms of the Creative Commons Attribution 4.0 International license.

### Using the miniTurbo-expressing HFFs to identify novel *Toxoplasma* PVM proteins.

**FIG 4:**
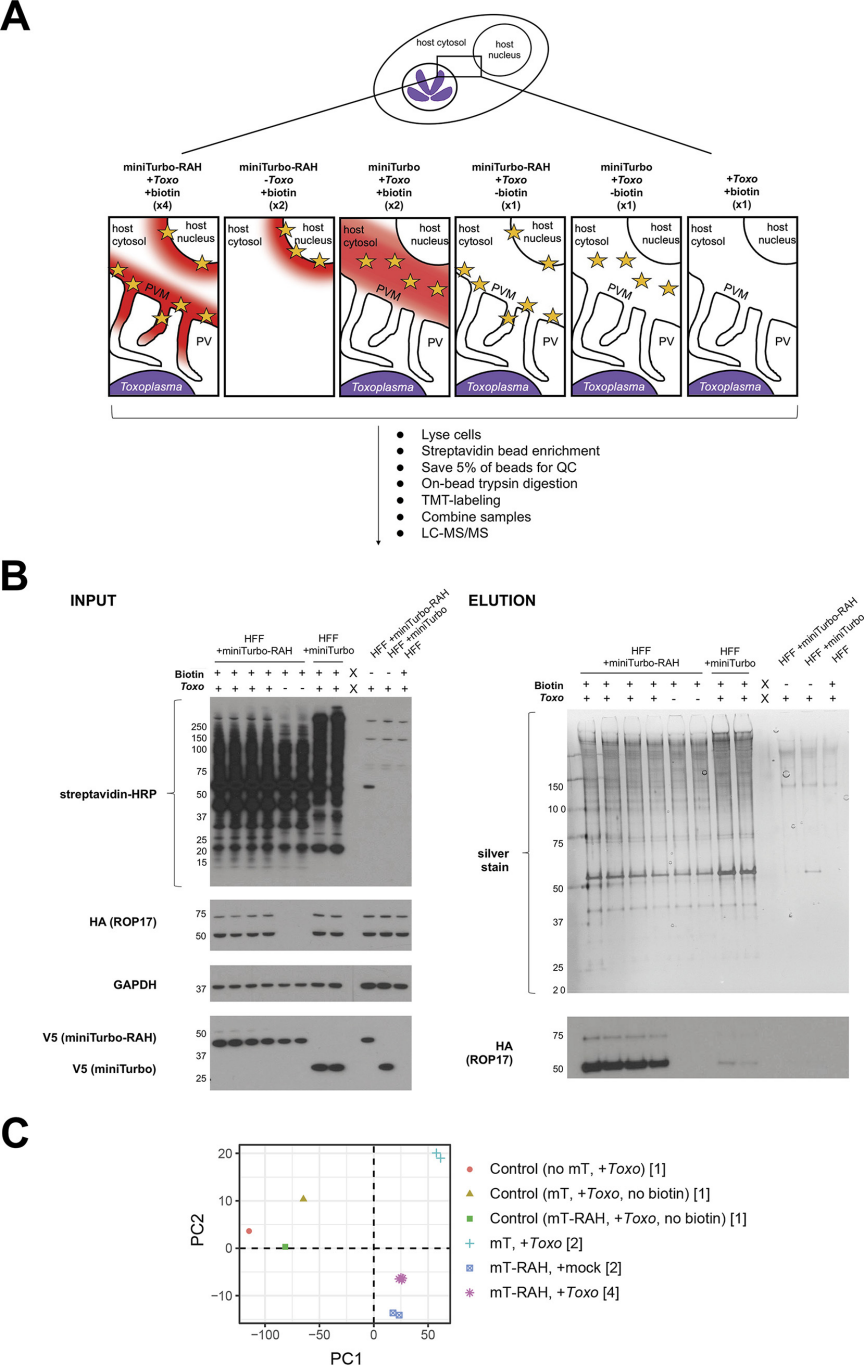
Quantitative mass spectrometry-based proteomic experimental setup and quality control checks. (A) Overview of proteomics experimental design and cartoon depiction of expected regions of biotinylation (depicted by red cloud) near the *Toxoplasma* PVM based on miniTurbo fusion localization (yellow star) and +/– biotin in the 11 samples submitted for LC/MS-MS. The number of replicates for each sample is indicated in parentheses below the description. The indicated samples were infected with RH*Δrop17*::ROP17-3xHA parasites for 22 h and then treated with 50 μM biotin for 1 h. After treatment and biotin-labeling, cells were lysed, and biotinylated proteins were enriched with streptavidin magnetic beads; 5% of the beads were saved for a quality control check, the remaining protein was digested on-bead, and subsequent peptides were conjugated to TMT labels. All 11 samples were then combined and analyzed by LC-MS/MS. (B, INPUT) Western blot of whole-cell lysates. A sample of each whole-cell lysate depicted in panel A was run on an SDS-PAGE gel and blotted with streptavidin-HRP to visualize all biotinylated proteins, mouse anti-HA-HRP antibodies to visualize endogenous ROP17, and mouse anti-V5 antibodies to visualize miniTurbo expression. (ELUTION) Silver stain and Western blots of eluates from streptavidin affinity purification (from the 5% of beads saved prior to on-bead digestion). The beads were washed and proteins eluted from the beads by boiling in the presence of 2 mM biotin and BME. Equivalent volumes of each elution were run on an SDS-PAGE gel and stained with silver to visualize total protein material eluted from the beads. Equivalent volumes of each elution were additionally run on a separate SDS-PAGE gel and blotted with mouse anti-HA-HRP antibodies to check for specific enrichment of endogenous ROP17 in the PVM-miniTurbo localized samples (miniTurbo-RAH + *Toxo*, lanes 1–4). Approximate migration of a ladder of size standards (sizes in kDa) is indicated. (C) Principal-component analysis of the 11 samples analyzed in the proteomics experiment. Numbers in square brackets indicate the number of replicates for each sample type analyzed, as described in panel A.

To identify PVM-localized proteins using quantitative proteomics, the HFF cell lines were either mock-infected or infected with RHΔ*rop17*::ROP17-3xHA tachyzoites for 22 h, biotin was then added for an additional hour, and biotinylated proteins were enriched by affinity purification. Biotinylated proteins captured on the streptavidin beads were digested with trypsin, and the peptides from each experimental condition were then labeled with tandem mass tag (TMT) reagents, combined, and analyzed by liquid chromatography tandem mass spectrometry (LC-MS/MS) (Fig. 4A). Western blotting and silver staining from a reserved fraction of these samples showed that the washes were sufficient to remove most background protein binding and that the PVM-localized protein ROP17 was enriched in all four infected miniTurbo-RAH replicates over all other controls (Fig. 4B). Additionally, principal-component analysis of the proteomic data set showed strong clustering of the biological replicates, indicating high reproducibility (Fig. 4C). To quantify differences across experimental groups, we calculated log_2_ fold changes (log_2_FC) and *P* values (moderated *t* test) by aggregating all replicates. The full data set showing the results for all the proteins detected is presented in Data Set S1.

10.1128/mBio.00260-21.1 DATA SET S1 Complete quantitative mass spectrometry data. Download Data Set S1, XLSX file, 2.2 MB. Copyright © 2021 Cygan et al. 2021 Cygan et al. https://creativecommons.org/licenses/by/4.0/ This content is distributed under the terms of the Creative Commons Attribution 4.0 International license.

**FIG 5:**
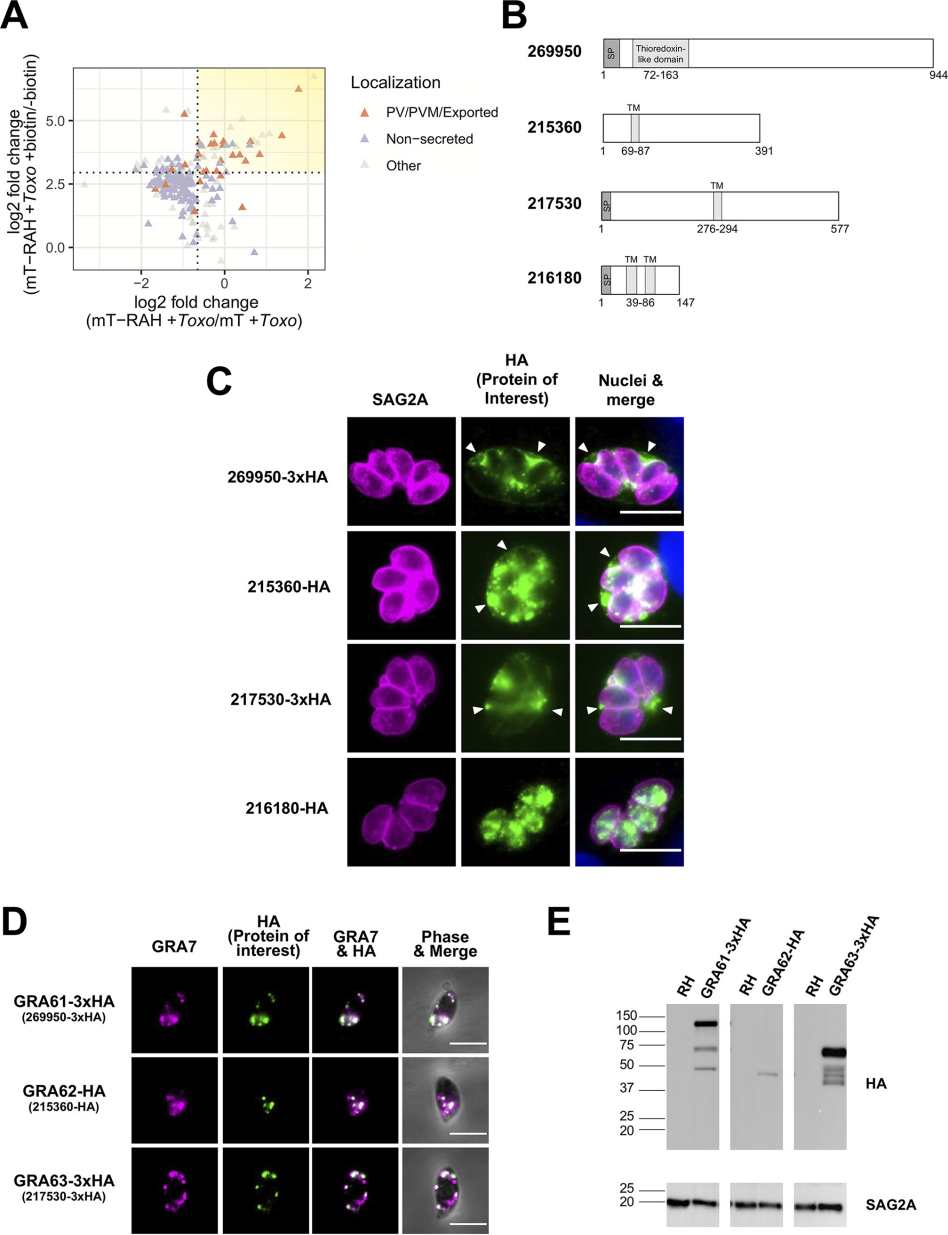
The newly identified *Toxoplasma* proteins 269950 (GRA61), 215360 (GRA62), and 217530 (GRA63) localize to the *Toxoplasma* parasitophorous vacuole in infected cells and derive from the dense granules. (A) Two-dimensional plot showing log_2_ fold changes between miniTurbo-RAH and miniTurbo-infected samples (*x* axis) and log_2_ fold changes between +biotin and –biotin miniTurbo-RAH samples (*y* axis). *Toxoplasma* proteins known to be exported or PV/PVM-localized or putatively nonsecreted (see Materials and Methods) are indicated by orange and purple symbols, respectively. Dotted lines indicate the optimal threshold for separation of known PV/PVM/exported versus nonsecreted *Toxoplasma* proteins. Yellow shading indicates the position on the plot of the proteins of interest described in Table 1. (B) Schematic representations of the indicated proteins showing predicted N-terminal signal peptides (SP), predicted transmembrane domains (TM), and predicted homology to conserved domains from ToxoDB.org (version 53). (C) Representative immunofluorescence microscopy images of endogenously tagged HA-protein (269950-3xHA and 217530-3xHA) or protein ectopically expressed under its native promoter (215360-HA and 216180-HA). The tachyzoite single clones expressing HA-tagged proteins of interest were allowed to infect HFFs for 20 h before the infected monolayers were fixed with methanol. The corresponding tagged proteins were detected with rat anti-HA antibodies (green), while all tachyzoites were detected with rabbit anti-SAG2A antibodies (magenta). Host and parasite nuclei were stained with DAPI (blue). Arrowheads indicate vacuoles with HA-staining outside the parasites. Scale bar is 10 μm. (D) Representative immunofluorescence microscopy images of free tachyzoites expressing endogenously HA-tagged protein (269950-3xHA and 217530-3xHA) or protein ectopically expressed under its native promoter (215360-HA). Staining was with rabbit anti-GRA7 antibodies (magenta) and rat anti-HA antibodies (green) with the data superimposed on phase contrast images of the parasites themselves in the right-most panels. Colocalization is apparent as white coloring in the merged images. Scale bar is 5 μm. (E) Western blotting of HA-tagged proteins. Lysates of HFFs infected with parasite single clones expressing GRA61/62/63 were resolved by SDS-PAGE, blotted, and probed with rat anti-HA antibodies to detect the HA-tagged proteins. Rabbit anti-SAG2A staining was used as a loading control for total parasite protein. Approximate migration of a ladder of size standards (sizes in kDa) is indicated.

We refined the candidate PVM-localized proteins by comparison to lists of likely positives (PV/PVM-localized or exported *Toxoplasma* proteins based on published literature) and likely negatives (putatively nonsecreted *Toxoplasma* proteins based on a recent hyperLOPIT proteomics data set [25]). Optimal log_2_FC thresholds were calculated by receiver-operating characteristic (ROC) analysis for the miniTurbo-RAH + *Toxo*/miniTurbo-RAH + *Toxo* (no biotin) comparison (log_2_FC > 2.95) and for the miniTurbo-RAH + *Toxo*/miniTurbo + *Toxo* comparison (log_2_FC > −0.65) (Fig. S2). These optimized thresholds maximize true-positive rates while minimizing false-positive rates. Using these threshold criteria (Fig. 5A, yellow shading), we produced a high-confidence list of *Toxoplasma* proteins putatively enriched at the PVM (Table 1). Table 1 also provides information on the hyperLOPIT-derived predicted subcellular localization (25) for reference. Note that the hyperLOPIT data were derived using purified, extracellular tachyzoites, so no prediction can be made about the ultimate destination of these proteins within an infected host cell from these data alone.

**TABLE 1:** List of *Toxoplasma* proteins putatively enriched at the parasitophorous vacuole membrane^*a*^

Gene ID (TGGT1_)	Description	mT-RAH + biotin/mT-RAH-biotin log_2_ FC	Evidence of PV/PVM/exported localization within infected cells	Predicted subcellular localization within tachyzoites (hyperLOPIT)^*b*^
269950	Hypothetical protein (GRA61)	6.73	PV/PVM (this study)	Dense granules
220240	GRA31	6.23	PV/PVM (16)	Dense granules
231210	Sarcalumenin/eps15 family protein	5.06	NA	Nucleus-chromatin
279350	Hypothetical protein	4.73	NA	Dense granules
215360	Hypothetical protein (GRA62)	4.48	PV/PVM (this study)	Dense granules
310780	GRA4	4.44	PV/PVM (30)	Dense granules
216180	Hypothetical protein	4.42	No (this study)	ER
309590	ROP1	4.40	PV/PVM (62)	Rhoptries 1
217530	Hypothetical protein (GRA63)	4.21	PV/PVM (this study)	Dense granules
410370	MAF1-related	4.17	NA	NA
258580	ROP17	4.17	PV/PVM (12)	Rhoptries 1
210370	ROP54	4.13	PV/PVM (13)	Rhoptries 1
207920	Hypothetical protein	4.11	NA	Unknown
229010	RON4	4.10	PV/PVM (51)	Rhoptries 1
306030	Glutathione s-transferase, N-terminal domain-containing protein	4.07	NA	ER
203600	GRA50	4.07	PV/PVM (32)	Dense granules
249530	Putative exportin 1	4.06	NA	Cytosol
289530	Ribosomal protein RPL19	4.03	NA	60S ribosome
411470	MAF1-related	4.02	NA	NA
292130	Ribosomal protein RPL13A	4.02	NA	60S ribosome
215470	Ribosomal protein RPL10A	3.99	NA	60S ribosome
230980	Myosin I	3.97	NA	Unknown
311470	RON5	3.94	NA	Rhoptries 1
300100	RON2	3.91	NA	Rhoptries 1
286450	GRA5	3.68	PV/PVM (63)	Dense granules
270240	MAG1	3.67	PV/PVM (64)	Dense granules
254720	GRA8	3.64	PV/PVM (31)	Dense granules
258870B	Hypothetical protein	3.64	NA	NA
411430	ROP5	3.62	PV/PVM (29)	NA
306338B	Putative dynein gamma chain, flagellar outer arm	3.58	NA	NA
306060	RON8	3.56	Nascent PVM (50)	Rhoptries 1
208830	GRA16	3.42	PV/exported (65)	Dense granules
220950	MAF1b	3.40	PV/PVM (27)	Dense granules
314810	Ribosomal protein RPL7	3.31	NA	60S ribosome
226430	Reticulon protein	3.19	NA	ER
309120	Ribosomal protein RPL4	3.05	NA	60S ribosome
215220	GRA22	3.01	PV/PVM (66)	Dense granules
254470	MYR1	3.00	PV/PVM (67)	Dense granules
291930	RNA recognition motif-containing protein	2.96	NA	Cytosol
288650	GRA12	2.96	PV/PVM (68)	Dense granules

a The relative abundance of each protein was determined across all samples using the peptide-level TMT quantification, and the log_2_ fold change (log_2_ FC) was determined for various sample ratios. The proteins were filtered by a minimum log_2_ FC of 2.95 for the (mT-RAH + *Toxo*)/(mT-RAH + *Toxo* no biotin) ratio and a log_2_ FC of −0.65 for the (mT-RAH + *Toxo*)/(mT + *Toxo*) ratio. Displayed here are all the *Toxoplasma* proteins (identified by at least 2 unique peptides, with a protein score of >25, and with protein coverage of >1%) which passed the above thresholds, ranked according to the log_2_ FC between the averaged (mT-RAH + *Toxo*)/(mT-RAH + *Toxo* no biotin) samples, the majority *Toxoplasma* identifiers (TGGT1_; i.e., the proteins that contain at least half of the peptides belonging to a group of proteins that cannot be unambiguously identified by unique peptides), and the descriptive name for each protein (“Description” column). If the protein had been previously localized to the PV/PVM or exported (via microscopy), the reference is indicated. NA, No microscopy evidence of PV/PVM localization or no predicted subcellular localization within tachyzoites.

b The predicted subcellular localization of these proteins within tachyzoites based on a recent hyperLOPIT proteomics study is included for reference (25). The hypothetical proteins of unknown location and function that were chosen for follow-up localization studies are bolded.

10.1128/mBio.00260-21.4 FIG S2 Analyses to determine optimal cutoff thresholds based on likely positives and likely negatives. (A) Receiver operating characteristic (ROC) curve showing the true-positive rate (TPR) and false-positive rate (FPR) for classification of known PV/PVM/exported-localized and putatively nonsecreted *Toxoplasma* proteins (see Materials and Methods) at all log_2_ fold-change values ([mT-RAH + *Toxo*]/[mT-RAH + *Toxo*, no biotin]). The optimal point (maximum value of Youden’s index, defined as max*[J = sensitivity + specificity − 1]*) was identified as 2.95. (B) Normalized density plot showing the frequency of known PV/PVM-localized and putatively nonsecreted proteins across the log_2_ fold-change values ([mT-RAH + *Toxo*]/[mT-RAH + *Toxo*, no biotin]). The dotted line indicates the optimal threshold as identified by ROC analysis in panel A. (C) ROC curve generated as in panel A at all log_2_ fold-change values ([mT-RAH + *Toxo*]/[mT + *Toxo*]). The optimal point (Youden’s index) was identified as −0.65. (D) Normalized density plot showing the frequency of known PV/PVM-localized and putatively nonsecreted proteins across the log_2_ fold-change values ([mT-RAH + *Toxo*]/[mT + *Toxo*]). The dotted line indicates the optimal threshold as identified by ROC analysis in panel C. Download FIG S2, PDF file, 0.1 MB. Copyright © 2021 Cygan et al. 2021 Cygan et al. https://creativecommons.org/licenses/by/4.0/ This content is distributed under the terms of the Creative Commons Attribution 4.0 International license.

As expected, we observed strong enrichment of several dense granule and rhoptry proteins known to be PVM-localized and therefore exposed to the host cytosol, including ROP1, MAF1b- and MAF1-related proteins, ROP17, ROP54, GRA5, and ROP5 (12, 13, 26–29). Additionally, many proteins (such as GRA31, GRA4, GRA8, and GRA50) which had previously been shown to be secreted into the PV (16, 30–32), and predicted by Phobius (33) to contain transmembrane domains, suggesting integration into the PVM and exposure to the host cytosol, were also enriched. Importantly, we did not observe enrichment of abundant parasite proteins present within the PV lumen, such as GRA1 or SAG1/2, consistent with the effective labeling radius of the activated biotinyl-5-AMP intermediate being quite small (estimated 10 nm [34]) and thus unlikely to extend far beyond the PVM.

In addition to these known examples of PVM-localized proteins, several uncharacterized (hypothetical) proteins were also enriched, including TGGT1_269950, TGGT1_215360, TGGT1_216180, and TGGT1_217530, suggesting a likely PVM localization. Consistent with this, all four were predicted by hyperLOPIT (25) to enter the secretory pathway, with 269950, 215360, and 217530 being predicted dense granule proteins, while 216180 was predicted to be located in the endoplasmic reticulum (ER). All four proteins also possessed features, including either predicted N-terminal signal peptides or transmembrane domains, that were also consistent with potential secretion into the PV/PVM (Fig. 5B). Thus, these four proteins were chosen for subsequent analysis of their localization in infected cells.

To localize these candidate PVM proteins in infected cells, we isolated single clones of parasites in which these genes were endogenously modified to encode a 3×HA tag immediately before the stop codon or in which a C-terminally hemagglutinin (HA)-tagged version of these proteins was ectopically expressed under each gene’s native promoter. These genomic modifications were verified by PCR, and protein localization was then assessed by IFA. The results (Fig. 5C) showed a clear PV-like signal outside the parasites in the 269950-3×HA, 215360-HA, and 217530-3×HA clones, including at the periphery of the PV. Thus, we conclude that 269950, 215360, and 217530 are at least transiently localized to the *Toxoplasma* PV/PVM during infection. In contrast, we did not observe any staining outside the parasites in the clone expressing 216180-HA, suggesting that, at least at this time point, this protein is either not present in the PVM and is thus a false positive in our enrichment or it is present at the PVM at too low a level to be detected by IFA; its designation by hyperLOPIT as an ER protein is consistent with these results.

To confirm their putative designation as dense granule proteins based on hyperLOPIT data, we examined the subcellular localization of the three newly identified PVM proteins in free tachyzoites (because it was not found to be at the PVM, we did not further investigate the localization of 216180). The results (Fig. 5D) showed that all three colocalize by IFA with a known dense granule protein, GRA7, and we therefore designate these and refer to them here as GRA61 (269950), GRA62 (215360), and GRA63 (217530).

Two of these three new GRA proteins, GRA61 and GRA63, have a consensus TEXEL motif (i.e., the three-amino acid sequence RRL) that in many GRA proteins is cleaved by the parasite’s ASP5 protease (35). To make an initial assessment as to whether this or other proteolytic processing is in fact occurring, we analyzed the mobility of the HA-tagged proteins by SDS-PAGE and Western blotting. The results (Fig. 5E) showed that the mobility of the tagged proteins was within the approximate range expected for the primary translation product (after removal of the N-terminal signal peptide predicted for GRA61 and GRA63), suggesting that these proteins might not be further cleaved; i.e., GRA61, GRA62, and GRA63 are predicted to have mature molecular masses of 100, 43, and 61 kDa versus their mobility in SDS-PAGE-indicated sizes of about 120, 45, and 65 kDa, respectively. Consistent with this, cumulative mass spectrometry data for GRA63 from several studies available on ToxoDB.org (version 54) shows 24 spectral counts for peptides that span the RRL cleavage site (i.e., amino acid 328), with no counts detected for a peptide that would be produced by cleavage at this site. Together, the gel mobility and mass spectrometry data strongly argue against cleavage at this site in GRA63. For GRA61, on the other hand, there were no peptides reported on ToxoDB that span its RRL (i.e., amino acid 112), but neither were there any peptides that would result from ASP5 cleavage at this site. There were, however, over 400 peptides detected from elsewhere in the protein, indicating that the protein itself is readily detected. Given that ASP5 cleavage would change the molecular mass of GRA61 by only ∼8% (100 kDa for the uncleaved protein versus 92 kDa for a product cleaved at the RRL), gel mobility is of limited utility in addressing the possibility of such processing, so further work, e.g., using mobility of the protein harvested from wild-type and ASP5-deleted mutants, will be needed to directly address this question.

### Using the miniTurbo-expressing HFFs to identify novel human PVM proteins.

**FIG 6:**
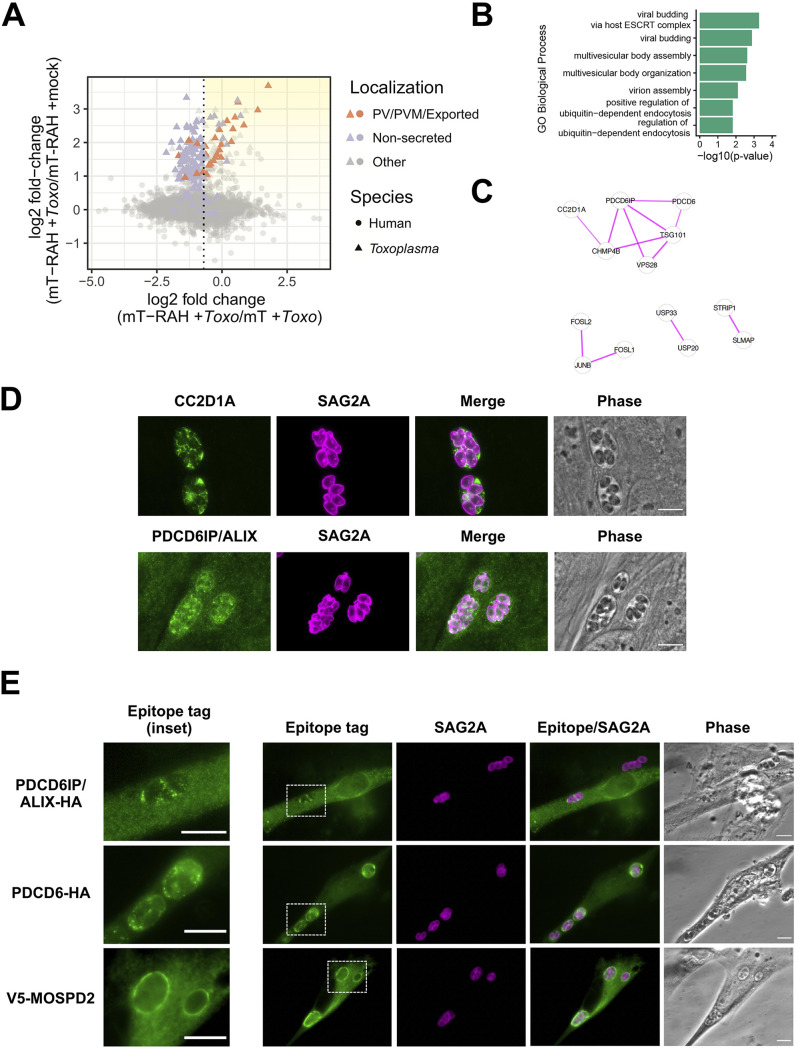
Host ESCRT-associated proteins PDCD6, ALIX, and CC2D1A, and the host ER-organelle tethering protein MOSPD2, localize to the *Toxoplasma* parasitophorous vacuole membrane. (A) Two-dimensional plot showing log_2_ fold changes between miniTurbo-RAH and miniTurbo-infected samples (*x* axis) and log_2_ fold changes between infected and mock-infected miniTurbo-RAH samples (*y* axis). *Toxoplasma* proteins known to be exported or PV/PVM-localized or putatively nonsecreted are labeled using the same color scheme as in Fig. 5A. The dotted line indicates the optimal threshold for separation of known PV/PVM/exported-localized proteins and nonsecreted *Toxoplasma* proteins. Yellow shading indicates the position on the plot of the proteins of interest described in Table 2. (B) Significant terms from functional enrichment analysis performed on the candidate host PVM proteins from Table 2. Terms are shown for the Gene Ontology biological process functional database. (C) Result of STRING version 11 (61) protein-protein interaction analysis of the candidate host PVM proteins from Table 2 with singletons (proteins with no association to another protein) removed. The thickness of the lines between proteins indicates the degree of confidence of the interaction. (D) Representative immunofluorescence microscopy images of the localization of the host proteins CC2D1A and PDCD6IP/ALIX during *Toxoplasma* infection. HFFs were infected with RH parasites for 20 h before the infected monolayers were fixed with methanol. Rabbit anti-CC2D1A antibodies and rabbit anti-ALIX antibodies were used to detect the corresponding host proteins (green). Tachyzoites were detected with rabbit anti-SAG2A antibodies (magenta), and the infected monolayer was visualized with phase microscopy. Scale bar is 10 μm. (E) Representative immunofluorescence microscopy images of the localization of host proteins upon transient overexpression in HFFs. HFFs were transiently transfected with plasmids expressing the indicated tagged proteins and then infected with RH tachyzoites 24 h posttransfection. The parasites were allowed to infect for 16 h before the monolayers were fixed with methanol. Mouse anti-V5 and rat anti-HA antibodies (green) were used to detect the corresponding host proteins. Tachyzoites were detected with rabbit anti-SAG2A antibodies (magenta), and the infected monolayer was visualized with phase microscopy. Dashed white boxes indicate the PVs expanded in the insets (left-most panels). Scale bars are 10 μm.

In addition to novel parasite proteins, we also aimed to identify host protein candidates at the PVM by focusing on the host proteins enriched upon infection (miniTurbo-RAH + *Toxo*/miniTurbo-RAH + mock) (Fig. 6A). First, the list of candidate host proteins was filtered using the log_2_FC threshold established by ROC analysis for the miniTurbo-RAH + *Toxo*/miniTurbo + *Toxo* comparison (log_2_FC > −0.65). Since we could not generate similar lists of likely positive and likely negative host proteins at the PVM due to the lack of knowledge on host proteins at this interface, we instead rank-sorted the host proteins by their log_2_FC upon infection (Fig. 6A, yellow shading). The top 50 enriched host proteins are presented in Table 2. This list of proteins was analyzed for functional enrichment (Fig. 6B), which revealed “viral budding via host ESCRT (endosomal sorting complexes required for transport) complex” to be the most significantly enriched Gene Ontology biological process term. Further analysis looking for known protein-protein interactions (Fig. 6C) confirmed an enrichment for several proteins known to physically associate within the ESCRT complex—CC2D1A, PDCD6, PDCD6IP, TSG101, VSP28, and CHMP4B (Fig. 6C) (36).

**TABLE 2:** List of the top 50 human proteins putatively enriched at the *Toxoplasma* parasitophorous vacuole membrane^*a*^

Accession no.	Description	Gene	mT-RAH + *Toxo*/mT-RAH + mock log_2_ FC
Q5VSL9	Striatin-interacting protein 1	STRIP1	2.13
O75340-2	**Isoform 2 of programmed cell death protein 6**	**PDCD6**	**2.00**
A0A0C4DH65	Protein KRBA1	KRBA1	1.72
Q8NHP6	**Motile sperm domain-containing protein 2**	**MOSPD2**	**1.70**
Q6P1N0	**Coiled-coil and C2 domain-containing protein 1A**	**CC2D1A**	**1.67**
Q6N043	Zinc finger protein 280D	ZNF280D	1.66
P49750	Isoform 4 of YLP motif-containing protein 1	YLPM1	1.53
Q5T0F9	Coiled-coil and C2 domain-containing protein 1B	CC2D1B	1.51
A6NMQ1	DNA polymerase	POLA1	1.46
Q6P1N0-2	**Isoform 2 of coiled-coil and C2 domain-containing protein 1A**	**CC2D1A**	**1.41**
Q6PJI9	GATOR complex protein WDR59	WDR59	1.39
Q99816	Tumor susceptibility gene 101 protein	TSG101	1.37
Q7Z460	CLIP-associating protein 1	CLASP1	1.18
Q99570	Phosphoinositide 3-kinase regulatory subunit 4	PIK3R4	1.18
Q99661	Kinesin-like protein KIF2C	KIF2C	1.16
Q9NYA1-2	Isoform 2 of sphingosine kinase 1	SPHK1	1.13
P21580	Tumor necrosis factor alpha-induced protein 3	TNFAIP3	1.12
Q07960	Rho GTPase-activating protein 1	ARHGAP1	1.05
Q8NDZ4	Deleted in autism protein 1	DIPK2A	0.97
Q9P273	Teneurin-3	TENM3	0.96
Q9Y2K6	Ubiquitin carboxyl-terminal hydrolase 20	USP20	0.95
Q8TEY7	Ubiquitin carboxyl-terminal hydrolase 33	USP33	0.85
P15408	Fos-related antigen 2	FOSL2	0.84
P17275	Transcription factor jun-B	JUNB	0.78
P15407	Fos-related antigen 1	FOSL1	0.75
Q8WUM4	**Programmed cell death 6-interacting protein**	**PDCD6IP**	**0.72**
Q14BN4	Sarcolemmal membrane-associated protein	SLMAP	0.71
Q9UK41	Vacuolar protein sorting-associated protein 28 homolog	VPS28	0.67
Q86VY9	Transmembrane protein 200A	TMEM200A	0.67
P18850	Cyclic AMP-dependent transcription factor ATF-6 alpha	ATF6	0.65
Q6UWP8	Suprabasin	SBSN	0.64
O94822-3	Isoform 3 of E3 ubiquitin-protein ligase listerin	LTN1	0.64
O75154-3	Isoform 3 of Rab11 family-interacting protein 3	RAB11FIP3	0.62
P53985	Monocarboxylate transporter 1	SLC16A1	0.62
Q96F4	Interleukin-17 receptor A	IL17RA	0.62
Q9NU22	Midasin	MDN1	0.59
Q96JA1	Leucine-rich repeats and immunoglobulin-like domains protein 1	LRIG1	0.57
P35475	Alpha-l-iduronidase	IDUA	0.50
O95983	Methyl-CpG-binding domain protein 3	MBD3	0.49
Q5D862	Filaggrin-2	FLG2	0.48
Q9NYI0	PH and SEC7 domain-containing protein 3	PSD3	0.48
P32189	Glycerol kinase	GK	0.46
Q9BTX7	Alpha-tocopherol transfer protein-like	TTPAL	0.45
P53794	Sodium/myo-inositol cotransporter	SLC5A3	0.44
P30048	Thioredoxin-dependent peroxide reductase mitochondrial	PRDX3	0.43
Q9C0C4	Semaphorin-4C	SEMA4C	0.43
P40189	Interleukin-6 receptor subunit beta	IL6ST	0.43
P29590-2	Isoform PML-5 of Protein PML	PML	0.43
P02786	Transferrin receptor protein 1	TFRC	0.42
Q9H444	Charged multivesicular body protein 4b	CHMP4B	0.42

a The relative abundance of each protein was determined across all samples using the peptide-level TMT quantification, and the log_2_ fold change (log_2_ FC) was determined for various sample ratios. The proteins were first filtered by requiring a minimum log_2_ FC of −0.65 for the (mT-RAH + *Toxo*)/(mT + *Toxo*) ratio and a log_2_ FC of 0 for the (mT-RAH + *Toxo*/mT-RAH + *Toxo*, no biotin) ratio (see Materials and Methods). Displayed here are the top 50 human proteins (identified by at least 2 unique peptides, with a protein score of >25, and with a protein coverage of >1%) ranked according to the log_2_ FC between the averaged mT-RAH + *Toxo* samples versus mT-RAH + mock samples. Also shown are their majority human UniProt identifiers (“Accession Number” column), i.e., the proteins that contain at least half of the peptides belonging to a group of proteins that cannot be unambiguously identified by unique peptides, the descriptive name for each protein (“Description” column), and the gene symbol (“Gene” column). The proteins of unknown location in infected cells chosen for follow-up localization studies are bolded.

Host PDCD6IP (also known as ALIX) has previously been reported to associate with at least the nascent PV during the time of *Toxoplasma* invasion (37), as well as the only host protein enriched in a BioID experiment looking to identify proteins within the *Toxoplasma* PV (16). For these reasons, we wanted to determine if this and the other ESCRT-related host proteins were associated with the PVM at later time points in infection. To do this, we first assessed the localization of PDCD6IP/ALIX and CC2D1A during infection with *Toxoplasma* tachyzoites via IFA using antibodies raised against the endogenous human proteins. The results (Fig. 6D) showed clear staining of CC2D1A and PDCD6IP/ALIX surrounding the PVs, as well as present in the spaces between parasites, likely within the IVN that often fills such regions. To confirm and extend these results, we also assessed the localization of PDCD6IP/ALIX and PDCD6 (also known as ALG-2), a Ca^2+^ binding protein that interacts with several ESCRT accessory proteins (38), in infected host cells transiently overexpressing C-terminally HA-tagged versions of these proteins. The results (Fig. 6E, top two rows) showed moderate association of PDCD6/ALIX to the PV/PVM and very strong association of PDCD6/ALG-2 to the PV/PVM.

Another compelling PVM-recruited host candidate that was highly enriched was MOSPD2, an ER-anchored membrane protein that has recently been shown to function as a scaffold to mediate interactions with organelle-bound proteins to form contact sites between the ER and endosomes, mitochondria, or Golgi (39). To determine if MOSPD2 is recruited to the PVM during infection, we assessed its localization in host cells transiently overexpressing an N-terminally V5-tagged version of MOSPD2. The results (Fig. 6E, bottom row) showed very strong association of MOSPD2 to the PVM. As discussed further below, this could be a clue to the mechanism by which host ER associates with the PVM.

## DISCUSSION

The *Toxoplasma* PVM is a critical platform for host-parasite interactions, and by using miniTurbo-catalyzed proximity-labeling, we identified candidate host and parasite proteins localized at the host cytosolic side of this interface and validated several (GRA61 [269950], GRA62 [215360], GRA63 [217530], PDCD6IP/ALIX, PDCD6, CC2D1A, and MOSPD2) as indeed PV/PVM-localized.

The functional role of the three newly identified GRA proteins is not yet completely known, but they could play a part in the many interactions occurring at the PVM. All three are predicted to be “dispensable” for growth *in vitro* based on a genome-wide CRISPR screen for genes that impact fitness under *in vitro* growth conditions (40). This is true of a majority of known PVM proteins and is consistent with their role interfacing the immune system which is not in play *in vitro*. GRA63, but not GRA61 and GRA62, was assessed in a CRISPR screen examining genes necessary for growth during the acute phase of a mouse infection and found to be dispensable under these conditions as well (41). This was also true of at least one other GRA known to interact with the immune system, GRA25 (42), so this does not preclude an important biological role for GRA63.

GRA61 was identified in a different CRISPR screen for genes important for growth in the peritoneum of infected mice (43) and, subsequently, in activated macrophages *in vitro* (44). Interestingly, in the latter study, parasites deleted for *GRA61* showed increased tendency for vacuoles to be coated with an immunity-related GTPase, Irgb6, when infecting murine macrophages stimulated with IFN-γ *in vitro*. The precise role for GRA61 has not been determined, but its thioredoxin-like domain was speculated to potentially be involved as a sulfhydryl oxidase and, possibly, protection against oxidative stress (43). Our identification of GRA61 as a PV/PVM-localized protein provides a further clue to how this protein functions.

A very exciting aspect of these data set is the new hypotheses that it creates regarding novel host-parasite interactions at the PVM. We confirmed that several host ESCRT accessory proteins are recruited to the PVM during infection, but this leaves unanswered the question of the biological significance of this association. Future work determining whether these host proteins are functioning in a pro- or anti-*Toxoplasma* manner will be critical to understanding how important this recruitment is to parasite development (e.g., how does knockout/knockdown of these components affect parasite replication?). ESCRT proteins mediate many functions within the cell related to membrane remodeling (36, 45), many of which could be relevant to *Toxoplasma*. For example, ESCRT-III (one of the ESCRT subcomplexes) can deform membranes, assemble into long filaments on negatively curved membranes, and promote vesicle budding (36). CC2D1A is a regulator of the ESCRT-III protein CHMP4B (46), and both proteins were highly enriched at the PVM in our data set. These results suggest the interesting hypothesis that host ESCRT machinery is promoting or maintaining the *Toxoplasma* IVN. Of note, a very recent report (47) confirms that host ESCRT machinery is operating at the PVM and that this is key to the uptake of host proteins into the parasites. GRA14 was shown to mediate this ESCRT recruitment, but we did not detect this protein in our own experiments reported here, but as discussed further below, the failure to detect a given protein can be due to many reasons and on its own has little predictive value.

Another interesting hypothesis suggested by our data is that the human protein MOSPD2 may mediate the recruitment of host ER to the PV, given its normal function in bringing the ER in close apposition to other host membranes (39). MOSPD2 interacts with other proteins via FFAT motifs (two phenylalanines [FF] in an acidic track [AT]), and analyses assessing the role of *Toxoplasma* proteins possessing such motifs may yield interesting candidates for parasite PVM proteins that may be interacting with MOSPD2. Future immunoprecipitation experiments using infected cells expressing tagged MOSPD2 will be necessary to identify potential parasite binding proteins and further test this hypothesis.

Additionally, future variations of this approach would help to generate even more information as to what proteins are present at the PVM under different conditions. For example, one could assess if there are differences in the proteins present at this interface between different strains of *Toxoplasma*, or even in the closely related organism *Neospora*, and under different stress conditions (e.g., IFN-γ treatment). Moreover, combining these data with other recently developed complementary approaches will undoubtedly give us further insight into the molecular composition of the PVM (48).

While the proximity-labeling approach we used is extremely powerful, we would like to briefly discuss a few important limitations. The first is that the data are likely to be biased toward the most proximal miniTurbo-RAH interactors (i.e., those that are physically closest and spend the most time in the vicinity of the PVM-localized miniTurbo-RAH protein). Given that we used the PVM-localizing RAH domain from ROP17 for this study to bring miniTurbo in proximity to the PVM, it is possible that some of the most enriched *Toxoplasma* proteins identified are direct interactors with this portion of ROP17 (a kinase involved in both inactivation of host IFN-γ-induced immunity-related GTPases (IRGs) at the PVM as well as protein translocation across the PVM (12, 49)). Indeed, we found ROP5, a ROP17-interactor identified biochemically by tandem affinity purification studies of ROP17 (12), to be enriched within the infected HFF miniTurbo-RAH cells (Table 1). It is interesting then to consider the two most enriched parasite proteins, GRA61 and GRA31, as possible ROP17-interacting proteins. Consistent with such a possibility is the fact that Δ*gra61* parasites showed a decreased ability to fend off host IRGs at the PVM (44). A scenario where ROP17 associates with the PVM via binding to GRA61 would be one way to explain this result, but future work will be needed to dissect the precise nature and role of their interaction. Moreover, future experiments in Δ*gra61* and Δ*gra31* parasites will be necessary to determine if either play a role in protein translocation across the PVM.

Additionally, despite our efforts to use stringent washes (i.e., radioimmunoprecipitation assay [RIPA] buffer, 1 M KCl, 0.1 M Na_2_CO_3_, and 2 M urea) to minimize enrichment of nonbiotinylated proteins, it is important to note that secondary interactors could be enriched, as with any protein enrichment experiment. This includes proteins that are inherently sticky or strongly interact with other biotinylated proteins on the beads. Of note, components of the *Toxoplasma* moving junction (RON2/4/5/8), a complex present at the host cytosolic face of the nascent PVM (50), and at least one of which (RON4) is also present at the PV/PVM postinvasion (51), were strongly enriched in this experiment (Table 1). RON4 has also been shown to directly bind to human ALIX in recent biochemical experiments (37). Because in the protocol we used here, the biotinylated peptides themselves remain on the beads after on-bead trypsin digestion and thus are not subject to mass spectrometry, we cannot use these data alone to conclude whether any given protein was enriched due to direct biotinylation or simply via association with other biotinylated proteins in the lysate (e.g., RON4). Our follow-up IFA experiments for at least ALIX, however, confirmed that like PDCD6, CC2D1A, and MOSPD1, it is a bona-fide PV/PVM-localized protein in infected human cells, and its enrichment is not simply a consequence of its interaction with RON4 in the protein lysate.

Finally, our candidate list of PVM proteins is likely incomplete due to both the limitations of miniTurbo-catalyzed biotinylation and TMT-quantification sensitivity. The biotinyl-5-AMP reactive intermediate that is formed by miniTurbo can only react with primary amines (i.e., free lysines or N termini). Therefore, if a proximal protein does not have a lysine residue exposed to react with the reactive biotin intermediate, it will not be labeled, and we will not detect it. Moreover, even with the use of state-of-the-art MS instrumentation, limitations in the efficiency of biotin enrichment and instrument sensitivity make it possible that we did not observe host cytosol-exposed PVM proteins that are lowly abundant. Despite these potential limitations, however, this approach has identified many novel molecular players at the PVM.

Altogether, the results reported here demonstrate the power of proximity-labeling coupled with quantitative mass spectrometry to identify proteins exposed to the host cytosol at the *Toxoplasma* PVM, and this rich data set will enable multiple avenues of future inquiry into crucial host-parasite interactions.

## MATERIALS AND METHODS

### Parasite strains, culture, and infections.

All *Toxoplasma* tachyzoites used in this study are in the type I “RH” background, either RHΔ*hpt*Δ*ku80* (52) or RHΔ*rop17*::ROP17-3xHA (49). These tachyzoites, and all subsequently generated lines, were propagated in human foreskin fibroblasts (HFFs) cultured in complete Dulbecco’s modified Eagle medium (DMEM) supplemented with 10% heat-inactivated fetal bovine serum (FBS; HyClone, Logan, UT), 2 mM l-glutamine, 100 U/ml penicillin, and 100 μg/ml streptomycin at 37°C with 5% CO_2_. The HFFs were obtained from the neonatal clinic at Stanford University following routine circumcisions that are performed at the request of the parents for cultural, health, or other personal medical reasons (i.e., not in any way related to research). These foreskins, which would otherwise be discarded, are fully deidentified and therefore do not constitute human subjects research.

Prior to infection, parasites were scraped and syringe-lysed using a 27-gauge (G) needle, counted using a hemocytometer, and added to HFFs. Mock infection was done by first syringe-lysing uninfected HFFs, processing this in the same manner as done for the infected cells, and then adding the same volume of the resulting material as used for infections.

### Plasmid construction.

DNA sequences for all fusion constructs used in this study can be found in File S1. Briefly, all plasmids were constructed using standard molecular biology techniques. The pCDNA-V5-miniTurbo-NES (Addgene plasmid no. 107170) (18) was used to generate the pCDNA-V5-miniTurbo-NES-RAH plasmid. The RAH domain of ROP17 was amplified from *Toxoplasma* RH genomic DNA using forward primer 5′-AAGACCCTATTACCGTGATGGGAGGTTGTC-3′ and reverse primer 5′-TCAGCCTATCAAAGGCGGAACTACCGGTG-3′. The pCDNA-V5-miniTurbo-NES-RAH fusion plasmid was generated from this plasmid by overlap extension PCR, and a short linker (AAGGGCTCGGGCTCGACCTCGGGCTCGGGA) was introduced between the nuclear export signal (NES) and ROP17 RAH sequence. V5-miniTurbo and V5-miniTurbo-RAH were cloned into the pLenti-CMV-Puro plasmid (a gift from Jan Carette, Stanford University; Addgene plasmid no. 17452) using Gibson Assembly (New England Biolabs [NEB]).

10.1128/mBio.00260-21.2 FILE S1 DNA sequences of fusion proteins used in this study. Download File S1, DOCX file, 0.02 MB. Copyright © 2021 Cygan et al. 2021 Cygan et al. https://creativecommons.org/licenses/by/4.0/ This content is distributed under the terms of the Creative Commons Attribution 4.0 International license.

For *Toxoplasma* endogenous tagging plasmids, ∼1,500 to 3,000 bp of the 3′-coding sequence of each gene, up to but not including the stop codon, was amplified from RH genomic DNA and cloned into the pTKO2-HPT-3×HA plasmid (53) using Gibson Assembly (NEB).

For *Toxoplasma* ectopic expression plasmids, plasmids to ectopically express HA-tagged proteins off their native promoters were created by PCR amplification of the open reading frame of each gene, minus the stop codon, plus ∼2,000 bp upstream of the start codon to include the native promoter, followed by cloning into the pGRA-HPT-HA plasmid (54).

For human ectopic expression plasmids, full-length cDNA for each gene was amplified from a HeLa cDNA library (TaKaRa Bio) and inserted into a pCDNA expression plasmid modified with either a C-terminal HA or N-terminal V5 tag under the human cytomegalovirus (CMV) promoter using standard molecular biology techniques.

### Toxoplasma transfections.

Endogenous tagging plasmids and/or ectopic expression plasmids (with the gene of interest expressed off its native promoter) were transfected into *Toxoplasma* via electroporation using the Amaxa 4D Nucleofector (Lonza) system. Tachyzoites were mechanically released in phosphate-buffered saline (PBS), pelleted, and resuspended in 20 μl P3 primary cell Nucleofector solution (Lonza) with —to 25 μg DNA for transfection. After transfection, parasites were allowed to infect HFFs in DMEM in T25 flasks for 24 h, after which the medium was changed to complete DMEM supplemented with 50 μg/ml mycophenolic acid and 50 μg/ml xanthine for selection for the hypoxanthine-xanthine-guanine-phosphoribosyltransferae (HXGPRT or HPT) marker for 3 to 5 days.

### Mammalian cell culture and stable cell line generation.

All mammalian cell lines were propagated in complete Dulbecco’s modified Eagle medium (DMEM) supplemented with 10% heat-inactivated FBS, 2 mM l-glutamine, 100 U/ml penicillin, and 100 μg/ml streptomycin at 37°C with 5% CO_2_, unless otherwise noted.

For preparation of lentiviruses, HEK 293T cells in 10-cm dishes were transfected at ∼80% confluence with the lentiviral plasmid pLenti-CMV-Puro (Addgene plasmid no. 17452) containing the gene of interest (2 μg) and the lentiviral packaging plasmids pVSV-G, pΔ*VPR*, and pAdvant (gifts from Jan Carette, Stanford University) using FuGENE HD transfection reagent (Promega) according to manufacturer’s instructions. After 24 h, the medium was replaced with fresh medium. Approximately 48 h after transfection, the cell medium containing the lentivirus was harvested and filtered through a 0.45-μm filter and supplemented with 8 μg/ml protamine sulfate. To generate stable lines, HFFs in 6-well plates were then infected with the virus-containing medium (1 ml). The following day, the viral-containing medium was removed and replaced with fresh, antibiotic-free medium. The HFFs were allowed to recover for 48 h and then selected with medium containing 2 μg/ml puromycin for 3 days.

### Mammalian cell transient transfections.

HFFs were grown on glass coverslips to ∼80% confluence and subsequently transfected with Lipofectamine LTX reagent (Invitrogen) and 500 ng of each pCDNA plasmid (with the tagged gene of interest under the human cytomegalovirus promoter) according to the manufacturer’s instructions in antibiotic-free medium. Cells were incubated with the transfection reagent for ∼16 h, the medium was changed, and tachyzoites were added for another 24 h.

### Immunofluorescence assay of infected cells.

Infected cells grown on glass coverslips were fixed and permeabilized using 100% cold methanol for 10 min. These were then washed 3 times with PBS for 5 min and blocked using 3% (wt/vol) bovine serum albumin (BSA) in PBS for 1 h at room temperature (RT). HA was detected with rat monoclonal anti-HA antibody 3F10 (Roche), SAG2A was detected using rabbit polyclonal anti-SAG2A antibodies (55), V5 was detected with mouse anti-V5 tag monoclonal antibody (Invitrogen R960), human CC2D1A was detected with rabbit anti-CC2D1A antibodies (Sigma HPA005436), human PDCD6IP/ALIX was detected with rabbit anti-ALIX antibodies (a gift from Wesley Sundquist, University of Utah), and biotinylated proteins were detected with streptavidin Alexa-Fluor-488 (Invitrogen S32354). Primary antibodies were detected with goat polyclonal Alexa Fluor-conjugated secondary antibodies (Invitrogen). Primary and secondary antibodies were both diluted in 3% BSA in PBS. Coverslips were incubated with primary antibodies for 1 h at RT, washed, and incubated with Alexa-Fluor-conjugated secondary antibodies (Invitrogen) for 1 h at RT. Vectashield with DAPI stain (Vector Laboratories) was used to mount the coverslips on slides. Fluorescence was detected using wide-field epifluorescence microscopy, and images were analyzed using ImageJ/FIJI software (NIH).

### Immunofluorescence assay of extracellular parasites.

Tachyzoites were mechanically released from infected HFFs and allowed to attach to poly-l-lysine-treated coverslips for 2 h at 37°C. The coverslips were then washed once with PBS, fixed in cold methanol for 10 min, and blocked in 3% (wt/vol) BSA in PBS for 1 h at RT. Localization of HA-expressing GRA61, GRA62, and GRA63 was assessed with rat monoclonal anti-HA antibody as described above, and localization of the known dense granule protein, GRA7, was assessed with rabbit anti-GRA7 antibodies (56). Primary antibodies were detected with Alexa-Fluor conjugated secondary antibodies (Invitrogen). Vectashield with DAPI stain (Vector Laboratories) was used to mount the coverslips on slides. Fluorescence was detected using wide-field epifluorescence microscopy and images were analyzed using ImageJ/FIJI software (NIH).

### Gels and Western blots.

Cell lysates were prepared at the indicated time points postinfection in Laemmli sample buffer (Bio-Rad) supplemented with 355 mM 2-mercaptoethanol (BME). The samples were boiled for 5 min, separated on a Bolt 4 to 12% Bis-Tris gel (Invitrogen), and transferred to polyvinylidene difluoride (PVDF) membranes. Membranes were blocked with 5% (wt/vol) nonfat dry milk in Tris-buffered saline (TBS) supplemented with 0.5% Tween 20, and proteins were detected by incubation with primary antibodies diluted in blocking buffer followed by incubation with secondary antibodies (raised in goat against the appropriate species) conjugated to horseradish peroxidase (HRP) and diluted in blocking buffer. HA was detected using an HRP-conjugated HA antibody (Roche 12013819001), V5 was detected with mouse anti-V5 tag monoclonal antibody (Invitrogen R960), SAG2A was detected using rabbit polyclonal anti-SAG2A antibodies (55), MAF1b was detected using rabbit polyclonal anti-MAF1b antibodies (27), GAPDH was detected using mouse monoclonal anti-GAPDH antibody 6C5 (Calbiochem), and biotinylated proteins were detected using streptavidin-HRP (Invitrogen S911). HRP was detected using an enhanced chemiluminescence (ECL) kit (Pierce). Silver-stained gels were generated using the Pierce silver stain kit (Thermo Scientific).

### Protein biotinylation.

Biotin labeling in HFFs was initiated with the addition of 50 μM biotin (Sigma B4501; from a 100 mM stock dissolved in dimethyl sulfoxide [DMSO]) for 1 h and terminated by cooling cells to 0°C on wet ice and washing away excess biotin (3 to 5 washes with ice-cold PBS). Then, either the cells were fixed for subsequent staining for IFA, lysates were prepared for Western blot analysis (resuspended in cold RIPA and clarified with a 10,000 × *g* spin for 10 min at 4°C), or clarified lysates were subjected to enrichment of the biotinylated proteins by streptavidin bead enrichment. To enrich for biotinylated proteins, the lysates were incubated with prewashed Pierce streptavidin-coated magnetic beads (Thermo Fisher 88817), rotating overnight at 4°C. The beads were subsequently washed 2 times with 1 ml of RIPA lysis buffer, 1 time with 1 ml of 1 M KCl, 1 time with 1 ml of 0.1 M Na_2_CO_3_, 1 time with 1 ml of 2 M urea in 10 mM Tris-HCl (pH 8.0), and 3 times with 1 ml RIPA lysis buffer. With the exception of the samples submitted for LC-MS/MS analysis, for which a detailed protocol is included below, the biotinylated proteins were eluted from the beads by incubation with Laemmli sample buffer supplemented with BME (Bio-Rad) and 2 mM biotin at 90°C for 10 min.

### Sample preparation for proteomics.

First, 8 × 10^6^ HFFs were plated in 11 15-cm dishes. Then, 24 h later, the HFFs were either infected with 12 × 10^6^ RHΔ*rop17*::ROP17-3xHA tachyzoites or mock-infected with an equivalent volume of syringe-lysed uninfected HFFs. Next, 22 h postinfection, the medium was replaced with either medium containing 50 μM biotin for 1 h to initiate biotin labeling or with fresh medium containing no added biotin. After 1 h of labeling, the cells were washed on wet ice 4 times with 20 ml of ice-cold PBS; 1 ml of RIPA lysis buffer supplemented with complete protease inhibitor cocktail (cOmplete, EDTA-free [Roche]) and phosphatase inhibitor cocktail (PhosSTOP [Roche]) was added to each dish, and the cells were scraped into the lysis buffer and transferred into microcentrifuge tubes. The lysates were then syringe-lysed 3 times with a 27-G needle and subsequently clarified with a 10,000 × *g* spin for 10 min at 4°C. The supernatant was collected, and the total protein concentration was measured using the Pierce BCA protein assay kit (Thermo Scientific).

For enrichment of biotinylated material, 3,500 μg of each protein lysate was added to 250 μl of streptavidin-coated magnetic beads (prewashed 5 times with RIPA buffer) and allowed to incubate with rotation overnight at 4°C. The beads were subsequently washed 3 times with 1 ml of RIPA lysis buffer (10 min each), 1 time with 1 ml of 1 M KCl (10 min), 1 time with 1 ml of 0.1 M Na_2_CO_3_ (1 min), 1 time with 1 ml of 2 M urea in 10 mM Tris-HCl pH 8.0 (1 min), and 3 times with 1 ml RIPA lysis buffer (10 min). The beads were transferred to a fresh microcentrifuge tube prior to the final wash in RIPA buffer. Then, 5% of the beads were removed and saved for quality control analysis of the enrichment. The protein from the 5% of beads was eluted by boiling the beads in Laemmli sample buffer supplemented with BME (Bio-Rad) and 2 mM biotin at 90°C for 10 min. The remaining beads in RIPA lysis buffer were shipped overnight on ice to the Carr Laboratory (Broad Institute) for subsequent analysis by LC-MS/MS.

### On-bead trypsin digestion of biotinylated peptides.

On-bead trypsin digestion was performed as described previously (18). The biotin-labeled proteins bound to magnetic beads were further washed to remove detergent traces. Magnetic beads were immobilized and washed twice with 200 μl of 50 mM Tris HCl buffer (pH 7.5) followed by two washes with 2 M urea/50 mM Tris (pH 7.5) buffer. A partial trypsin digestion was performed to release proteins from the beads by using 80 μl of 2 M urea/50 mM Tris containing 1 mM dithiothreitol (DTT) and 0.4 μg trypsin (mass spectrometry grade, Promega) for 1 h at 25°C. Magnetic beads were immobilized, and the supernatant containing partially digested proteins was transferred to a fresh tube. The beads were washed twice with 60 μl of 2 M urea/50 mM Tris buffer (pH 7.5), and the washes were combined with the on-bead digest supernatant. Proteins were reduced with 4 mM DTT for 30 min at 25°C with shaking, followed by alkylation with 10 mM iodoacetamide for 45 min in the dark at 25°C. Proteins were completely digested by adding 0.5 μg of trypsin and incubating overnight at 25°C with shaking. Following the overnight incubation, samples were acidified to 1% formic acid (FA) and desalted using stage tips containing 2× C_18_ discs (Empore) as described next. The stage tip column was conditioned with 1 × 100 μl methanol, 1 × 100 μl 50% acetonitrile (ACN)/1% FA, and 2 × 100 μl 0.1% FA washes. Acidified peptides were bound to the column, washed with 2 × 100 μl 0.1% FA, and eluted with 50 μl 50% ACN/0.1% FA. Eluted peptides were dried to using a vacuum concentrator.

### TMT labeling and fractionation of peptides.

Desalted peptides were labeled with 11-plex TMT reagents (Thermo Fisher Scientific) and fractionated as described previously (18). Dried peptides were reconstituted in 100 μl of 50 mM HEPES, labeled using 0.8 mg of TMT reagent in 41 μl of anhydrous acetonitrile for 1 h at room temperature. The TMT reactions were quenched with 8 μl of 5% hydroxylamine at room temperature for 15 min with shaking, and the labeled peptides were desalted on C_18_ stage tips as described above. Peptides were fractionated by basic reverse phase using styrenedivinylbenzene-reverse phase sulfonate (SDB-RPS; Empore) material in stage tip columns. Two-disc punches were packed in a tip and equilibrated with 50 μl methanol, 50 μl 50% CAN/0.1% FA, and 2 × 75 μl 0.1% FA washes. Peptides were reconstituted in 0.1% FA and loaded into the column, followed by conditioning with 25 μl of 20 mM ammonium formate (AF). Next, peptides were sequentially eluted into 6 × 100-μl fractions with 20 mM AF and various concentrations of ACN—5%, 10%, 15%, 20%, 30%, and 55%. The six peptide fractions were dried by vacuum centrifugation.

### Liquid chromatography and mass spectrometry.

Desalted peptides were resuspended in 9 μl of 3% ACN/0.1% FA and analyzed by online nanoflow liquid chromatography tandem mass spectrometry (LC-MS/MS) using a Proxeon Easy-nLC 1200 coupled to a Q Exactive HF-X hybrid quadrupole-Orbitrap mass spectrometer (Thermo Fisher Scientific). A 4-μl of sample from each fraction was separated on a capillary column (360 × 75 μm, 50°C) containing an integrated emitter tip packed to a length of approximately 25 cm with ReproSil-Pur C_18_-AQ 1.9-μm beads (Dr. Maisch GmbH). Chromatography was performed with a 110-min gradient of solvent A (3% ACN/0.1% FA) and solvent B (90% ACN/0.1% FA). The gradient profile, described as min:% solvent B, was 0:2, 1:6, 85:30, 94:60, 95:90, 100:90, 101:50, and 110:50. Ion acquisition was performed in data-dependent MS2 (ddMS2) mode with the following relevant parameters: MS1 acquisition (60,000 resolution, 3E6 AGC [automatic gain control] target, 10 ms max injection time) and MS2 acquisition (loop count = 20, 0.7 m/z isolation window, 31 NCE [normalized collision energy], 45,000 resolution, 5E4 AGC target, 105 ms max injection time, 1E4 intensity threshold, 15-s dynamic exclusion, and charge exclusion for unassigned, 1 and >6).

### Protein quantification.

Collected RAW LC-MS/MS data were analyzed using Spectrum Mill software package version 6.1 prerelease (Agilent Technologies). MS2 spectra were extracted from RAW files and merged if originating from the same precursor or within a retention time window of ± 60 s and *m/z* range of ± 1.4, followed by filtering for a precursor mass range of 750 to 6,000 Da and sequence tag length of >0. An MS/MS search was performed against a custom concatenated FASTA database containing (i) the human UniProt protein database downloaded in December 2017, (ii) a list of known common contaminants, and (iii) the Toxoplasma gondii protein database downloaded from ToxoDB.org (version 43). Search parameters were set to “Trypsin allow P,” <5 missed cleavages, fixed modifications (cysteine carbamidomethylation and TMT11 on N-term and internal lysine), and variable modifications (oxidized methionine, acetylation of the protein N terminus, pyroglutamic acid on N-term Q, and pyro carbamidomethyl on N-term C). Matching criteria included a 30% minimum matched peak intensity and a precursor and product mass tolerance of ± 20 ppm. Peptide-level matches were validated if found to be below the 1.0% false-discovery rate (FDR) threshold and within a precursor charge range of 2 to 6.

A protein-centric summary containing TMT channel intensities was exported from Spectrum Mill for downstream analysis in the R environment for statistical computing. Proteins were filtered to remove those that do not originate from humans or *Toxoplasma* and those with fewer than 2 unique peptides identified. Missing TMT intensity values were imputed assuming left-censored missing data by drawing values from the 1st percentile of all intensities. TMT ratios were calculated using the median of all TMT channels, followed by log_2_ transformation. Two-sample moderated *t* tests were performed using the limma package with correction for multiple testing by calculating local FDR and *q* values (57). The source code for the proteomics data analysis is available via a GitHub repository (https://github.com/pierremj/toxoplasma-proteomics).

### ROC threshold analysis.

Receiver-operating characteristic (ROC) analysis was performed in R using the rocit and plotROC libraries. A list of known PV/PVM/exported *Toxoplasma* proteins were used as likely positives, while a list of predicted nonsecreted *Toxoplasma* proteins (i.e., annotated as 19S/20S proteosome, 40S/60S ribosome, apicoplast, cytosolic, ER, mitochondrial, nuclear, plasma membrane, inner membrane complex, or tubulin cytoskeleton localized from a recent *Toxoplasma* LOPIT proteomics data set [25], and not containing a predicted signal peptide, and not annotated as “hypothetical”) were used as likely negatives. Both lists can be found in Data Set S1. The log_2_ fold change values ([mT-RAH + *Toxo*/mT + *Toxo*] and [mT-RAH + *Toxo*/mT-RAH + *Toxo*, no biotin]) were used as classifiers, and the optimal threshold points were determined using the Youden index method.

### Data analysis to generate tables.

To generate Table 1, the *Toxoplasma* proteins from Data Set S1 were filtered based on protein score of >25, protein coverage of >1%, mT-RAH/mT log_2_ FC of >−0.65 (from ROC analysis), and mT-RAH + *Toxo*/mT-RAH + *Toxo* (no biotin) log_2_ FC of >2.95 (from ROC analysis). All the *Toxoplasma* proteins that met these criteria are displayed in Table 1.

To generate Table 2, the human proteins from Data Set S1 were filtered based on protein score of >25, protein coverage of >1%, mT-RAH/mT log_2_ FC of >–0.65 (from ROC analysis), mT-RAH + *Toxo*/mT-RAH + *Toxo* (no biotin) log_2_ FC of >0, and adjusted *P* value of <0.01 (to remove endogenously biotinylated proteins). The list of proteins was then rank-sorted by the log_2_ FC of the mT-RAH + *Toxo*/mT-RAH + mock comparison, with the top 50 displayed in Table 2.

### Functional enrichment analysis.

Functional enrichment analysis was performed using the gost function with default parameters from the gprofiler2 library in R (58).

### STRING network analysis.

Generation of the protein-protein interaction network was performed using StringApp (59) in Cytoscape (version 3.8.2) (60). The network was filtered to remove singletons (proteins with no association to another protein) and to only include interactions for which experimental evidence existed within STRING (version 11) (61) with high confidence (score, >0.7).

### Data availability.

The original mass spectra and the protein sequence database used for searches have been deposited in the public proteomics repository MassIVE (http://massive.ucsd.edu) and are accessible at ftp://massive.ucsd.edu/MSV000086833/.
