# Supplementary figures and images for: Proximity-Labeling Reveals Novel Host and Parasite Proteins at the Toxoplasma Parasitophorous Vacuole Membrane
Source: mBio. 2021 Nov 9;12(6):e00260-21. doi: 10.1128/mBio.00260-21 (PMC8576527; doi:10.1128/mBio.00260-21)

**INPUT**

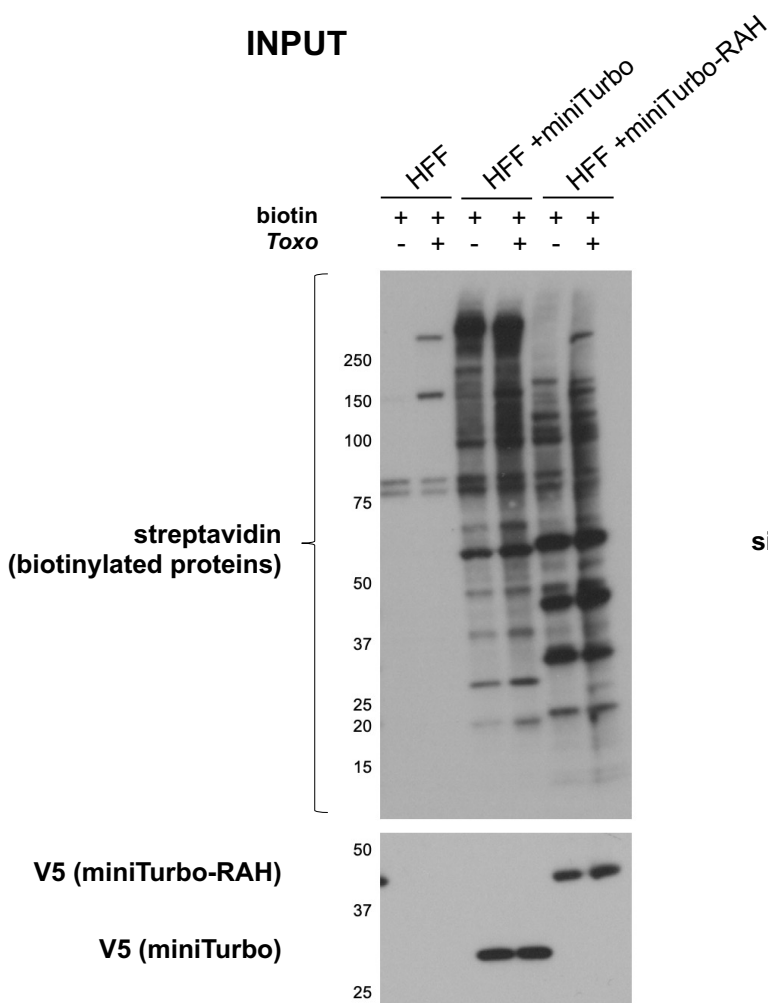

**ELUTION**

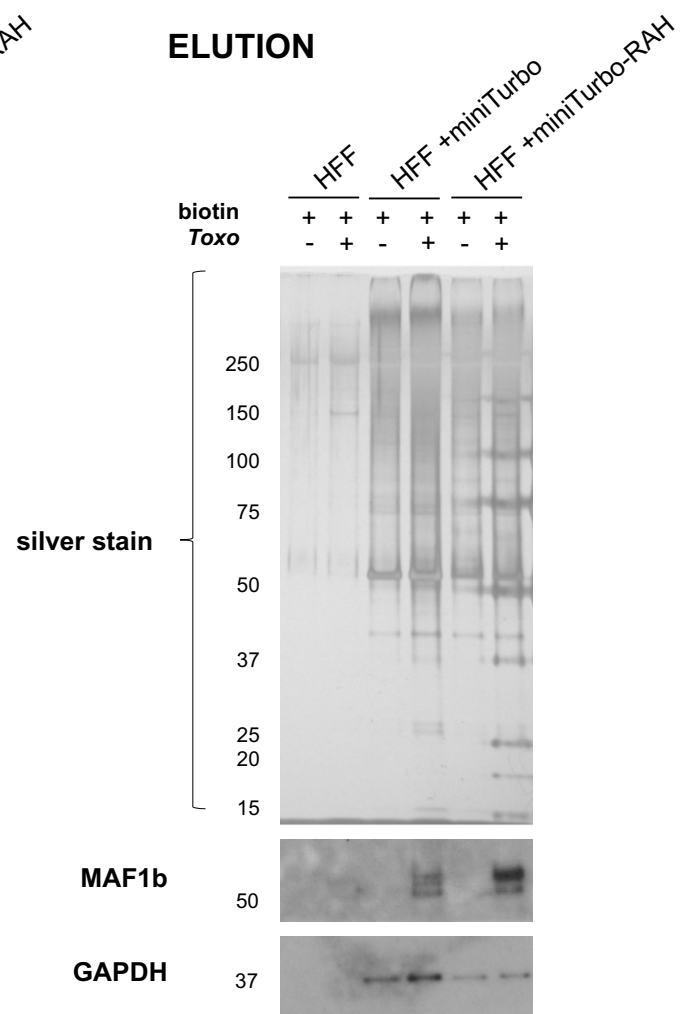

Supplement: FIG S1 [file mbio.00260-21-sf001.pdf]

**A**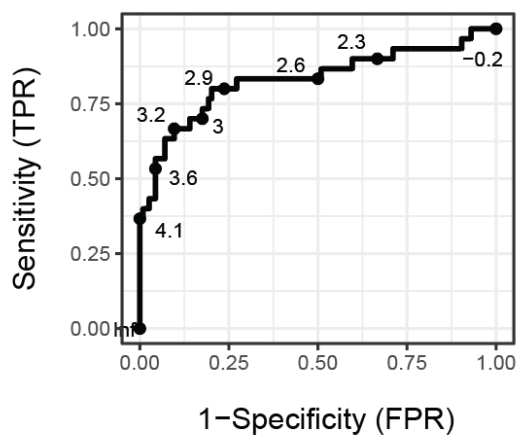**B**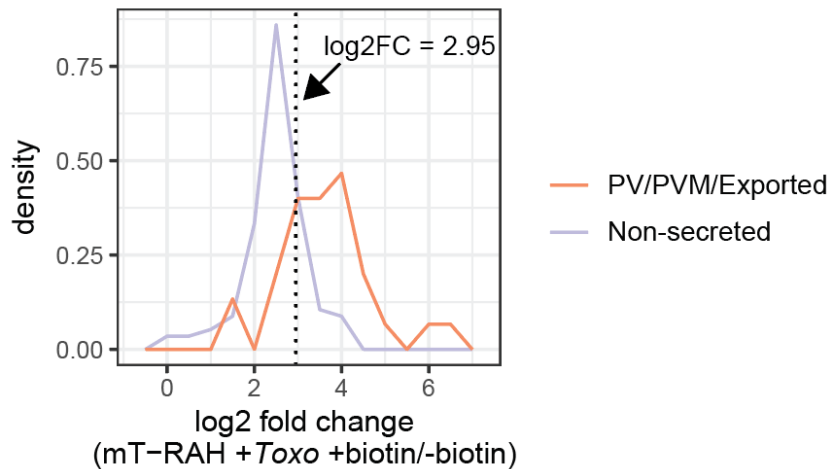**C**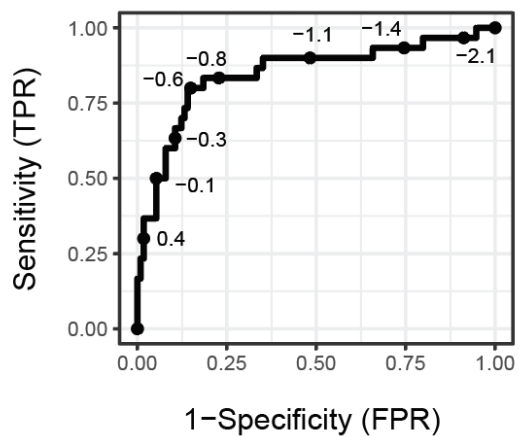**D**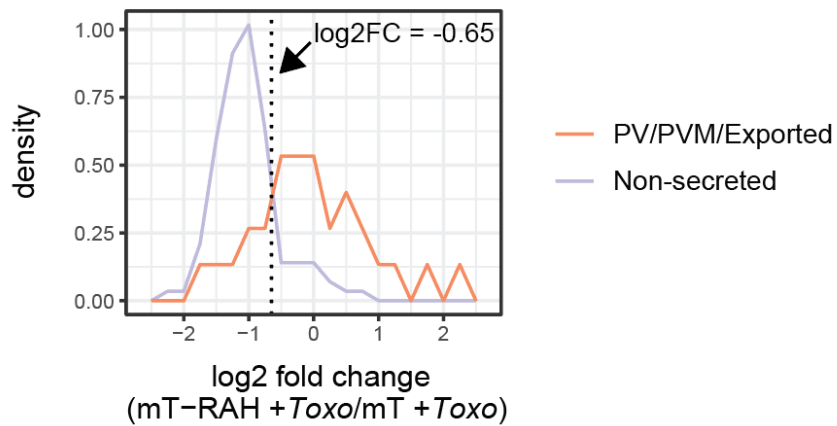

Supplement: FIG S2 [file mbio.00260-21-sf002.pdf]
