# Supplementary material for: Proximity-Labeling Reveals Novel Host and Parasite Proteins at the Toxoplasma Parasitophorous Vacuole Membrane
Source: mBio. 2021 Nov 9;12(6):e00260-21. doi: 10.1128/mBio.00260-21 (PMC8576527; doi:10.1128/mBio.00260-21)
Supplement: FILE S1 [file mbio.00260-21-s0001.docx]

**File S1.** DNA sequences of fusion proteins used in this study.

| **V5-miniTurbo-NES** |
| --- |
| GGCAAGCCCATCCCCAACCCCCTGCTGGGCCTGGACAGCACCGCTAGCATCCCGCTGCTGAACGCTAAACAGATTCTGGGACAGCTGGACGGCGGGAGCGTGGCAGTCCTGCCTGTGGTCGACTCCACCAATCAGTACCTGCTGGATCGAATCGGCGAGCTGAAGAGTGGGGATGCTTGCATTGCAGAATATCAGCAGGCAGGGAGAGGAAGCAGAGGGAGGAAATGGTTCTCTCCTTTTGGAGCTAACCTGTACCTGAGTATGTTTTGGCGCCTGAAGCGGGGACCAGCAGCAATCGGCCTGGGCCCGGTCATCGGAATTGTCATGGCAGAAGCGCTGCGAAAGCTGGGAGCAGACAAGGTGCGAGTCAAATGGCCCAATGACCTGTATCTGCAGGATAGAAAGCTGGCAGGCATCCTGGTGGAGCTGGCCGGAATAACAGGCGATGCTGCACAGATCGTCATTGGCGCCGGGATTAACGTGGCTATGAGGCGCGTGGAGGAAAGCGTGGTCAATCAGGGCTGGATCACACTGCAGGAAGCAGGGATTAACCTGGACAGGAATACTCTGGCCGCTATGCTGATCCGAGAGCTGCGGGCAGCCCTGGAACTGTTCGAGCAGGAAGGCCTGGCTCCATATCTGTCACGGTGGGAGAAGCTGGATAACTTCATCAATAGACCCGTGAAGCTGATCATTGGGGACAAAGAGATTTTCGGGATTAGCCGGGGGATTGATAAACAGGGAGCCCTGCTGCTGGAACAGGACGGAGTTATCAAACCCTGGATGGGCGGAGAAATCAGTCTGCGGTCTGCCGAAAAGCTGCAGCTGCCTCCCCTGGAGCGCCTGACCCTGGACTAA |
| **V5-miniTurbo-NES-linker-ROP17 RAH** |
| ATGGGCAAGCCCATCCCCAACCCCCTGCTGGGCCTGGACAGCACCGCTAGCATCCCGCTGCTGAACGCTAAACAGATTCTGGGACAGCTGGACGGCGGGAGCGTGGCAGTCCTGCCTGTGGTCGACTCCACCAATCAGTACCTGCTGGATCGAATCGGCGAGCTGAAGAGTGGGGATGCTTGCATTGCAGAATATCAGCAGGCAGGGAGAGGAAGCAGAGGGAGGAAATGGTTCTCTCCTTTTGGAGCTAACCTGTACCTGAGTATGTTTTGGCGCCTGAAGCGGGGACCAGCAGCAATCGGCCTGGGCCCGGTCATCGGAATTGTCATGGCAGAAGCGCTGCGAAAGCTGGGAGCAGACAAGGTGCGAGTCAAATGGCCCAATGACCTGTATCTGCAGGATAGAAAGCTGGCAGGCATCCTGGTGGAGCTGGCCGGAATAACAGGCGATGCTGCACAGATCGTCATTGGCGCCGGGATTAACGTGGCTATGAGGCGCGTGGAGGAAAGCGTGGTCAATCAGGGCTGGATCACACTGCAGGAAGCAGGGATTAACCTGGACAGGAATACTCTGGCCGCTATGCTGATCCGAGAGCTGCGGGCAGCCCTGGAACTGTTCGAGCAGGAAGGCCTGGCTCCATATCTGTCACGGTGGGAGAAGCTGGATAACTTCATCAATAGACCCGTGAAGCTGATCATTGGGGACAAAGAGATTTTCGGGATTAGCCGGGGGATTGATAAACAGGGAGCCCTGCTGCTGGAACAGGACGGAGTTATCAAACCCTGGATGGGCGGAGAAATCAGTCTGCGGTCTGCCGAAAAGCTGCAGCTGCCTCCCCTGGAGCGCCTGACCCTGGACAAGGGCTCGGGCTCGACCTCGGGCTCGGGAAGACCCTATTACCGTGATGGGAGGTTGTCACCCGTTTTAGGTGTTCAGGAACGTAGGGGGCGCAGTGTTCATTCGTATCATGAAGAACCAGTTTCCTTTTTCGATCAGCGTGCTTTTGATGAGTATACCTTCAGAAGAAGATCACAGCTTCATAGACAACGGGCACGGGCCGGACTGCGCTCAAGAATAAAACAGAATGTGAGACGACTCTGGACATCAGCCAGAGGCGCAGTGCGCGGGTGGGGTAGGCGAGTACGACGAAAAATCGGGGATTTGTTCGTTGGGCACTTGATGCCCCAGCTGCGGAGGCTACGGTTTTGGGACCAGGGCCTCCCACCGGTAGTTCCGCCTTTGATAGGCTGA |
|  |
| **ALIX-HA** |
| ATGGCGACATTCATCTCGGTGCAGCTGAAAAAGACCTCAGAGGTGGACCTGGCCAAGCCGCTGGTGAAGTTCATCCAGCAGACTTACCCAAGCGGCGGGGAAGAGCAGGCCCAGTACTGCCGCGCGGCGGAGGAGCTCAGCAAGCTGCGCCGCGCCGCAGTCGGTCGTCCGCTGGACAAGCACGAGGGCGCGCTCGAGACGCTCCTGAGATATTATGATCAGATTTGTTCTATTGAACCCAAATTCCCATTTTCTGAAAATCAGATCTGCTTGACATTTACCTGGAAGGATGCTTTCGATAAAGGTTCACTTTTTGGAGGCTCTGTAAAACTGGCTCTTGCAAGCTTAGGATATGAAAAGAGCTGTGTGTTGTTCAATTGTGCAGCCTTAGCTAGCCAAATTGCAGCAGAACAGAACCTGGATAATGATGAAGGATTGAAAATCGCTGCTAAACATTACCAGTTTGCTAGTGGTGCCTTTTTACATATTAAAGAGACGGTTTTATCTGCCTTAAGTCGAGAGCCGACCGTGGACATATCTCCAGATACTGTTGGGACCCTCAGTCTTATTATGCTGGCACAGGCTCAAGAAGTATTTTTTTTAAAAGCCACAAGAGATAAAATGAAAGATGCCATCATAGCTAAATTGGCTAATCAGGCTGCAGATTATTTTGGTGATGCTTTCAAACAGTGTCAATACAAAGATACTCTCCCCAAGTATTTTTATTTCCAGGAGGTGTTCCCTGTCTTGGCTGCAAAGCACTGTATCATGCAGGCCAATGCTGAGTACCATCAGTCTATCCTGGCAAAACAGCAGAAGAAATTTGGAGAAGAAATTGCAAGGTTACAGCATGCAGCAGAACTGATTAAAACAGTGGCATCTCGCTATGATGAATATGTTAATGTGAAGGATTTTTCTGACAAAATCAATCGTGCCCTTGCTGCAGCAAAGAAGGATAATGACTTCATTTATCATGATCGAGTTCCAGACCTTAAAGATCTAGATCCTATTGGCAAAGCCACACTTGTGAAATCTACCCCGGTCAATGTACCCATCAGTCAGAAATTTACTGATCTGTTTGAGAAGATGGTTCCCGTGTCAGTACAGCAGTCTTTGGCTGCCTATAATCAGAGGAAAGCCGATTTGGTTAACAGATCAATTGCTCAGATGAGAGAAGCCACCACTTTGGCAAATGGGGTGCTAGCTTCCCTTAATCTTCCAGCAGCAATTGAAGATGTGTCTGGAGACACTGTACCTCAGTCTATATTGACTAAATCCAGATCTGTGATTGAACAGGGAGGCATCCAGACTGTTGATCAGTTGATTAAAGAACTGCCTGAATTACTGCAACGAAATAGAGAAATCCTAGATGAGTCATTAAGGTTGTTGGATGAAGAAGAAGCAACCGATAATGATTTAAGAGCAAAATTTAAGGAACGTTGGCAAAGGACACCATCCAATGAACTGTATAAGCCTTTAAGAGCAGAGGGAACCAACTTCAGAACAGTTTTAGATAAAGCTGTGCAGGCAGATGGACAAGTGAAAGAATGTTACCAGTCTCATCGTGACACCATCGTGCTTTTGTGTAAGCCAGAGCCTGAGCTGAATGCTGCCATCCCTTCTGCTAATCCAGCAAAGACCATGCAGGGCAGTGAGGTTGTAAATGTCTTAAAATCCTTATTGTCAAATCTTGATGAAGTAAAGAAGGAAAGAGAGGGTCTGGAGAATGACTTGAAATCTGTGAATTTTGACATGACAAGCAAGTTTTTGACAGCCCTGGCTCAAGATGGTGTGATAAATGAAGAAGCTCTTTCTGTTACTGAACTAGATCGAGTCTATGGAGGTCTTACAACTAAAGTCCAAGAATCTCTAAAGAAACAGGAGGGACTTCTTAAAAATATTCAGGTCTCACATCAGGAATTTTCAAAAATGAAACAATCTAATAATGAAGCTAACTTAAGAGAAGAAGTTTTGAAGAATTTAGCTACTGCATATGACAACTTTGTTGAACTTGTAGCTAATTTGAAGGAAGGCACAAAGTTTTACAATGAGTTGACTGAAATCCTGGTCAGGTTCCAGAACAAATGCAGTGATATAGTTTTTGCACGGAAGACAGAAAGAGATGAACTCTTAAAGGACTTGCAACAAAGCATTGCCAGAGAACCTAGTGCTCCTTCAATTCCTACACCTGCGTATCAGTCCTCACCAGCAGGAGGACATGCACCAACTCCTCCAACTCCAGCGCCAAGAACCATGCCGCCTACTAAGCCCCAGCCCCCAGCCAGGCCTCCACCACCTGTGCTTCCAGCAAATCGAGCTCCTTCTGCTACTGCTCCATCTCCAGTGGGGGCTGGGACTGCTGCGCCAGCTCCATCACAAACGCCTGGCTCAGCTCCTCCTCCACAGGCGCAGGGACCACCCTATCCCACCTATCCAGGATATCCTGGGTATTGCCAAATGCCCATGCCCATGGGCTATAATCCTTATGCGTATGGCCAGTATAATATGCCATATCCACCAGTGTATCACCAGAGTCCTGGACAGGCTCCATACCCGGGACCCCAGCAGCCTTCATACCCCTTCCCTCAGCCCCCACAGCAGTCTTACTATCCACAGCAGATCTACCCGTACGACGTCCCGGACTACGCGTAG |
|  |
| **PDCD6-HA** |
| ATGGCCGCCTACTCTTACCGCCCCGGCCCTGGGGCCGGCCCTGGGCCTGCTGCAGGCGCGGCGCTGCCGGACCAGAGCTTCCTGTGGAACGTTTTCCAGAGGGTCGATAAAGACAGGAGTGGAGTGATATCAGACACCGAGCTTCAGCAAGCTCTCTCCAACGGCACGTGGACTCCCTTTAATCCAGTGACTGTCAGGTCGATCATATCCATGTTTGACCGTGAGAACAAGGCCGGCGTGAACTTCAGCGAGTTCACGGGTGTGTGGAAGTACATCACGGACTGGCAGAACGTCTTCCGCACGTACGACCGGGACAACTCCGGGATGATCGATAAGAACGAGCTGAAGCAGGCCCTCTCAGGTTTCGGCTACCGGCTCTCTGACCAGTTCCACGACATCCTCATTCGAAAGTTTGACAGGCAGGGACGGGGGCAGATTGCCTTCGACGACTTCATCCAGGGCTGCATCGTCCTGCAGAGGTTGACGGATATATTCAGACGTTACGACACGGATCAGGACGGCTGGATTCAGGTGTCGTACGAACAGTACCTGTCCATGGTCTTCAGTATCGTAATCTACCCGTACGACGTCCCGGACTACGCGTAG |
|  |
| **V5-MOSPD2** |
| GCAAGCCCATCCCCAACCCCCTGCTGGGCCTGGACAGCACCGCTAGCATGGCAGAGAATCACGCCCAGAATAAAGCCAAGCTCATCTCTGAGACCCGGAGGAGGTTCGAAGCTGAGTATGTGACAGATAAGTCAGATAAATATGATGCACGTGATGTTGAAAGGCTACAACAAGATGATAACTGGGTTGAAAGTTACTTATCTTGGAGACATAATATTGTAGATGAAACACTGAAGATGCTCGATGAGAGTTTTCAGTGGAGGAAAGAAATTTCTGTCAATGACCTTAATGAATCCTCCATTCCCAGATGGTTATTGGAAATTGGTGTTATTTATCTCCATGGTTATGACAAAGAAGGTAACAAATTGTTCTGGATCAGGGTGAAGTATCATGTAAAAGACCAGAAAACCATATTGGACAAAAAGAAGCTCATAGCATTCTGGTTGGAACGTTATGCTAAGAGGGAAAATGGGAAACCTGTAACAGTGATGTTTGACCTGTCAGAAACTGGAATAAATAGCATTGACATGGACTTTGTACGCTTTATCATCAACTGCTTTAAGGTTTATTACCCTAAATACCTCTCAAAAATAGTGATCTTTGATATGCCTTGGTTAATGAATGCTGCTTTCAAAATTGTGAAAACCTGGCTTGGTCCAGAAGCAGTGAGCTTGTTGAAGTTTACAAGCAAAAATGAAGTCCAGGACTATGTCAGTGTAGAATACCTGCCTCCCCACATGGGTGGAACTGATCCTTTCAAGTATAGCTATCCACCACTAGTAGATGATGACTTCCAGACCCCACTGTGTGAGAATGGGCCTATTACCAGTGAGGATGAAACTTCAAGTAAAGAAGACATAGAAAGTGATGGCAAAGAAACATTGGAAACAATTTCTAATGAAGAACAAACACCTCTTCTTAAAAAGATTAACCCAACCGAATCTACTTCCAAAGCAGAAGAAAATGAAAAAGTTGATTCAAAAGTGAAAGCTTTCAAGAAACCATTGAGTGTATTTAAAGGCCCCTTACTACACATCAGCCCAGCAGAAGAACTGTACTTTGGAAGTACAGAATCCGGAGAGAAGAAAACCTTAATAGTGTTGACAAATGTAACTAAAAATATAGTGGCATTTAAGGTGAGAACAACAGCTCCAGAAAAATACAGAGTCAAGCCAAGCAATAGCAGCTGTGACCCGGGTGCATCAGTGGATATAGTTGTGTCTCCCCATGGGGGTTTAACAGTCTCTGCCCAAGACCGTTTTCTGATAATGGCTGCAGAAATGGAACAGTCATCTGGCACAGGCCCAGCAGAATTAACTCAGTTTTGGAAAGAAGTTCCCAGAAACAAAGTGATGGAACATAGGTTAAGATGCCATACTGTTGAAAGCAGTAAACCAAACACTCTTACGTTAAAAGACAATGCTTTCAATATGTCAGATAAAACCAGTGAAGATATATGTCTACAACTCAGTCGTTTACTAGAAAGCAATAGGAAGCTTGAAGACCAAGTTCAGCGTTGTATCTGGTTCCAGCAGCTGCTGCTTTCCTTAACAATGCTCTTGCTTGCTTTTGTCACCTCTTTCTTCTATTTATTGTACAGTTAA |
|  |
| **269950-3xHA**  ATGATAGGTCTCTTTTCTTCTCACTTTTTGAGGCGTCACAAAATGGCGGGTCGCGCGAGCGTCGTCGTCTCCCTGCTTTTCTCCGCTTCGCTGTGGTCTCTTCTCTCCCGCGTCGATGGCGCGCCTGCGAATCTATCCCCCGCGGCGCACCCGGCTGTCCCTGCGGTCATTTCTGAGCACCCGTTCGGCACAAGCCCCGTCCATATGGCGACGTCCGCGTCAGGTTTCCGCGAATTCGTCGCGCAACATCTGACGGCGGGGGACGTCGTCATCGTCCTGTTTTTCTCGCGGCACTGTCCGCACTGCATCCGCTTCAAGGTTACCTACCGCCGCCTGGCAGACCGGCTCGGCCAGCGCGTCAAGACCGAGAAGAACGGCGCCTTCTTCGCAGCCGTCGACGCGGGCGCCGCGCAAGTCACTGACCAGGAGGACTCCATCTGGGACATTCTGCGGGAGTTTCGTGTCTCCTTCATCCCCGACGTCCGCCTCCTGGTGCCTCTGCACCGAGTTAGGAGCGCGGCGGATGAGGACGGCGCGGAACTAAAGAACGAAAGTCCAGGAACCGCCAAGGAACTCGGCCCTGACTACGGCTCCCTCGCGGCGTGGACCCTTCAAGTCAGCCACCATGAAATGTCCGAAGACGGCATCGATACGCTTCTTCGCACGTACATTGAAACCACTGGACTCGACAAGCTCGAGGAAAACGACGAACAATTCTCAGAAAAAAGgtaaaaggtccacgcagtgattctgcgtatccacttgttcgcttcgtttccggaccctgtggttctacagacacgtatcgctctggtggtggtgtccatacagtgtgatgattccacatatagatatacatgtatgtatgtgtacgtgtatatgtatctgtaggctacgaagctatgttcgcagttcagcgcgttgcagtactcaatacatacattgtttggacatccctgtgtacctgggcgaatgtgggcacagctaatgtttttcgtcgacagagacagcggggccgtccactcctcgacgtcgtaggtgtacccggcgagtctgcgctgtgcgttctgcgtgcttcagACGTTCTCGTCAGGAGATTTTGGAGAGCAACTTGAAGCTGCTGGAGGCGGTGTGTCCAACGACAGAGTTTCCCCCCGCTCCTGTTGGGTCTCCTGTTCCAGCTCTGTGTCGCGGGGGCGAACGGTGGTCGCCAGATGCTGAAAGGGTGAGTGGCGAGACGCCTGGAAACCGTCTGCACGACGCCCTCGCCGTCTTTCTGCATGTGATGAGGAGGTGGATCGTGGCGTCCGACTCGAGTCTGCATCTGTCGCTGGAGAAGGAACTCGCGCTTGCCCGTTTCCTCGAACTCGCGCTGTACGCGTTGCCGGGGAAGGCCATCAAGGGCGCTATCTTCCGACTACTGCTGCACCTCCGGACCCTCGGTTCTGGTCTTCAGCCGCTGCCTCAAAGCTTGTTGTCTCGCGAGGAGTTCCACGCTGCACTGGAGAAAGCCAGCCGGATGGGCGAGGCGGGGGCAGACGAAAACGCTGCAGCTGAGACAGCGTCGACGGCTTGGACTGTGACTTCGAAGCCGCCCTCAGAGCCGCAGTCCGAAGTCGAGGGAGAACTCCCCTTTGTGTCTCTTCTGCGTAAAGACAACTGGGAGAAACGGCTGACCGACTTCAAGGTCGCGGACGTATCGGGCGCAGACGCGTTCCCGGGAGCGCAGCAGCCTGCGATGCGGCACTGCTGCACCGTTCTGTGCGGCGTGTGGACCTTGTTCCACGTCATCGCTGAAGGCTTGCAGACGCAGTTTCAGCGGAAAGTTCGTCTCGCCCGCGAACGAGGTGCTCCGCTCCCTCAGGCCCATGAGACTCAGAGACAAACCGACAAGAGCGAGACGGAGCACGCAGACCGCGAACGGCAGACAGAGATGTACTTGCTGTCTCAAGCTAGCGAAGAGGAGCAGACGCGAGCGGAGCAAGCAAGGAGCGGGAGAATCGACTTGAAGTTTCTAGGCCCCTACGACAGTTTGACCATGTTTGCGACTCAAAAGTACAAGGAACTGCTGCGCCAGGAAGAGCACGAACATCTTCACAGACAAGACAGAGACACACCCAACTTGCGCGGGGAGTCTAGACGCGAGACCGACGTCTCGTTCATGATCGAGGCTCTAGAGGAAACATTCGGTCCTGTGAAAAACAAGCAAGCTCTCAGCgtaagcaaaagtcagagagtcctggggaacgccctgtcgcggaggtcgtgggtccaggaaacaaccgcgagtctcagaactcgagtttcgatcttgagccagagcataggtctacgttccgctgcgcttccccaatgcagtgtaatgcctttttcaagcgagaccgtgaactacgtattttgtgcgtctctctctgttgtgttttgcatgctggacgcatgcgcgtgtctgttcttctccgtcagttgtctctgtgttgagttaggtaagccgttgactctactttcttctgaaccagcgtggagctgtgtgtttttccgaatcttttttcagGCTCTGGAGGAGGAGTTGCTCGTGGAGGAGCGACGGTCGATTCTCCCCGCGAATGTTGCGATGTCGTCGCTCCGGGATTGGCTTTTCTCCTTCTTCATGTGCGTCGCATGTCGCACGCATTTTCTCCAGTGTTTCGAGCAAGGATTTTACGGCCGCGAGCAAGTTACGCCGCCGACCGCTGTGAAGCAGGAGAGCCTGGCGGCGCTGGCGCAGAAGCGAATGGAGTTTCAGGGTGGTCTTTCTTCCTTCGTCGAGAAGCTGGGAGAAAAGTCGAACTCCTCCCCCTCCGCGGGTGCTTCTGGTGTCGCTGCGCGCCTGGCGGCAGGCGGCTCCCTTTCAGACGCAGAGCGCGAGATGCTGCTGTCGCATTTGCCAGCGAGTCTGTGGCTGTCAGGGGAGTTTTTCCGCGTGAGAGACCCTCTGGATGTTGCTGAGGAAGAAGAGAATCTGAGGAACTTTGCGCTGTGGCTGTGGCGACTCCACAACGCCGTAACAGTGAGAACAGCAGCGGAGGCGATCGTCGAGGTACTGGTCGATGAGACCCTGGACGTCTCGAGTGTGTCGAGGGCGCAGCCCAGCAACGCGACTCCCGGCTTCTCCGCAACTGGCGAGCCTCTGGGGATCGGTGAAATGGCCAAGCGTGGACTGGGAAGTGCGTACTTTCTGCATACAGATCCGCGGTGGCCTCCTGCTCAAGTTGCGTCCTGTCTGCGCGAAGATGCTGCGCCTTTCGAACTGGGTTTCGACTTCGTGGCGGCTCGCGGCGCGACGGACGCGGTGCACTTGTGCGAAAATGTGGACTTCTCTGGAGACTTCGACTTGGAAAAAATTCGCCAGTGGCTCCGAAAAACCTACTGGGGCTCGACTTGGGGGGAGACGGCCAGCGACGTGGACGAGGGGGCCTTGAGCAAGGCCAGGCTTCAGTCTCCAGGTTCCTCGCGCAGGGATGTAGAGGCCGACGACCAGGATATCTACCCGTACGACGTCCCGGACTACGCTGGCTATCCCTATGATGTGCCCGATTATGCGTATCCTTACGATGTTCCAGATTATGCCTTATGA  **215360-HA**  ATGGGTACCGACGATTGGGCAGCATATGCCGGTGGAATCGACACTGGATCTGTAACTATCCCTGAATACATTGGAGTGCATGAGGACGACATTTTGCCTAGTCCCGGTAGAGTTAGTGGAGAAGTCCATCTATCTTCTAGACTCTCATTTTCGAAAGGCATCTTGGCAGGAAACAAAGATGCTCGTACACAGAAGCTGAAGCAGCTGCTACGTTACGTTGCAGGAGCCGCTGTAATTATTGGGTTAGTTGCAATCGGCGTGAAAACAGCATCGGTGCTGCGAGAGGTTTCCGCCAGTCAGCGGGCTACAGACGAAGTGAAGAAGCAGCTTGAGAACGAGATTACCCTTGATCAGGCATACCTGGATCGTGCAAGCGACATTGAACACCGAACTGAACAAAAACGAGCACAGATCACGGAGCTGGGGAGTCACGTTCCGGATGAATCTGTCGATGAAACTCCCTCACGAAAGCGCAAAAGCAGGATCAAGAAGTTGAATAATATCAAAAACGCATTTGGGTCTCTGGGAAAGAACAAACGCAAGAAGCGTCAGCCATTAGAGGGGTCGCTTCCTCCCTCGGGCGACACTCAACAGCAAATGCGACCCGAAGTGAGGGCCTCATCCTCGTTGGCAGTAGCGATCTCTGATGAACTACAGCCGTCACAGCAGCAAATGCAACCCGCGTTGAAAGGCCCATCCTCGTGGAGGGCAGCGCTCTCTGATGACCAGCTGTCGTCACGTTTATCTGACGGTTCAGGACAATCTTCAGTTGCTGGTAGCCTACGAGACCCGTTTCGTGATGTTGTCGCTGCAATTGAAGATGAAATGAAACATGTCGAAGAGGAGGAGGTTCAGTGGCAAAAGAAGCACGAAAGCGTGTATAAGAAGTACAAGGAAACCATCGGAATGAGAAATCCCGAAAAAGGGCCTCATGGTCAAATGTGGAAGATGGCTTCAGAACTTCAAGTTCCGGGGACTCATGCTGATGCGAAGCAATGGATTGAATCGTCCAGTAGGAGGAATTCGCAGTTGGCCAAAGCATTTTTCCGCAGATACTTTTTTCCGGCAGCAACTACCGTGCTAAACGACAGAGAAGTTGACGCTCAGAAAGGAATAGCATCTGAGAGGGAAAAACTAAAAGAGCTAGAAAAAACCACGGGTAACCGCGATACAATCCCATGGTACCCGTACGACGTCCCGGACTACGCGTAG |
| **217530-3xHA**  ATGCCATCTTGGTGGCGGGTGTTCCCGCTATGCAACTTGCTGCTGCTTCTACTTTCCGGAGTTACGCAGCAATGCGGGCCTCgtaagcttgtatactgcacttcagggaaacaagaaaaataactggtgcatcaacttatatcggcgttgattccgcgcagagcttactgaaacggagcacccagcgttctccaatggagcaccattggtgccgtgattggacagcgtttgtatctcggacagacaccgacgtctcatgcgtgggttatgaaaatggcggttcttgcgttctccgtgtgctttcagGGCATGTATTAGGGTCCGAACAATTTGACGGGGGATGGCGTGCATACCCCTCGAAAAgtaagacttctcaaatcaaagtgcgagactgtcgctataaaacatctcaaatcgtcggatcgaaggagctgtacacagtctgttttgtgcgtgatgtggtgtttttggtctggcctgcagTTGACCGTTATGTTGCTCAAATGGAAAGATTTATCGAGAAGGAACCAGgtaggccatacgttaaagacggcgtgtcacaccatatgcttttagctattcgatgttaacatgacagttccttcccaccgaagacgagctcacatttgccgccgcctgttccgttcttgctgcattcgtgcagCGGGTGATCTGATTTCGTTCATCAGTGACAAAGCTGACCCGATCAATAAAAGAGAACTCATGTATGCCACCCTTATTTTCGGAAGGAAACATGGTGGTCCAGAAATAAGACCCGGAGTGAGGCACACGCTGACCTCTTTCTACCGAAGCCTCGCGACAAAGCTCAACATTCCCAAGTgtaagaaggcagagcatacgctgcactgaaacctgttggcagctgggtgtcgctttgacacttaaatccttgacagtgtaggccgtgcagtagcatgatatagtataaatttgtgctgtggactcactgtggatcatatttgtgtgtttggtgtcaatgcacgtggcacgagtttcactttgagaaaactgccgaaagcaccccagttaatagcgtattatggtagcgatgagaagctgcctgttcgttgttcattcgacctgagcttggagtacactgtcttgattgcggcacaatcgcagcctgtaccgtctacaggaagaacatacgaagtaaagtgactttcacagtttccatgttgttgattgaaatggaaagtttggctctttcaactgacctcggtgtacagtccaggtccattcgttttatcttgcttctggtattgcagCCGAGGTACAAAAAATGAAGGAAGAGGTGGCCAAGCAACTTTCTGCCGCAGTCGCAGAAAGGGACCTCGAACTGGAGAGAAAGAGACAGCACGTCCAAGAAGAACGGCAGTCTCGACAGAGATTGAGATATTCTTCTCATAAATTATCAACCGGCTCCTCTGCATCCCGTGGTCCTGAGGATGATGCCGTATTGCGTCCTCCGTCTCTATCCCCCACAACAGCTGATGTATTCGAGGATGGAGAAGACAGTGGGCACATGAGAGATGTGCCAGACAGCCTGCGACAGTCGCCCGCTCTTGGTACAAAAAACCTTGATGACGTTGAAAGCGGCGCGCGAAGTTCCAGCCGTGTTGGTCAGACGGGTAGACCTCCTCAAACAGGTGCCGAGTCACCGCCGGAGGGACCTAGCAGGCCATCCTATTTATCGGAGGGGTGGGCAGCCGTCCGTAAGCATAGGAAGGGAATCCTGGCGGCCGTTGCCGCAGCCGCGGTTGCTGCAGGTTTGTTTGGCGCAGTCAGGAGGATGAAACCATGGAAAGGAATAGCAGGGTCACGAGCATTTTCATGGAATGAATCGACATGCCGACTTGCGCTGAAGGTAGACATGGCGCGTCGCTTGAACGCAATGGGAGCAGTGGCCGATGTGGTCCGCGTTCAAGCAAATAATTCTCAAGGAAATCTTGAAGTGACCTATAGAGAGAAACCGGCTTTCGTCAGCTCAAAAGGAGGGCATTTGATTGATCCGATCAAATACTACCTTTCCCTAGGACATGCTTTGACAACTAGGAAAGAAGCATTCGCGGTTCCAACCAGTTGCGTAGCTTCCCGGTCCTCGACGTACCTAGTTACCAGGAATGGGAACGATATCAGCGGTGTCATCGTGGATTTGGATTTCCGTCCGGCACCATTTGCAGTCCCGAGAGAGGTTGTTACCAAAGAAGAAGTCGAGCATCTGTTACAAACAATGAGCGAATCCGACAGCGAATGGACCCTTTATTTCAACTGCACCCTCCATCTTACTGTTGCAAGCGATAGCTTGGTAGGACCGCCCGTTGTTCTAGTCGATCCTGCGATTGGAGCGGTAGAGCTTGGAAATAGTATCTCGCAGAACAAAACGTTTGAAATCCCCCTTCGCTGCCTAGCGCGTAATCCGAATGAGGTGAAATACATGTCTCTGGAACGGGATATCAATGGTACCAGCATGACTGACCTTATTTTGATCATGGAAGAAGCAGGTGACGCGGAGAGGGAGGCATGGACAGAAATCCCCAAGCGAGCCGACGTGCAAACCGGAGATGATATATCTTTTCTTTTAGAAGAGGAAAGAGACGACAAAAGTGCTGAAGCCTTGTTTAAGATCAACAGAATCTACCCGTACGACGTCCCGGACTACGCTGGCTATCCCTATGATGTGCCCGATTATGCGTATCCTTACGATGTTCCAGATTATGCCTTATGA |
| **216180-HA**  ATGATAACTGTTATTTTGTGTTCGGTGCTCTTCTATTCGTTGGGCCTTGTGGCCCCAACCTCGGCGGCATTGCATGCTTGCATTAAGCAGAAAACATTGCCCAACGAAAACGCGATGCTGCTGTGGACACAGTACTTCCTGCTTTTTTCTGCTCTCGTCACTGTTGTGTTTCCATACGTTGTGACTCCTCTTTTCTTCTTGCTGCCGTCCTGGCTACTTGCTATGGTGAAACTGGTTCTAGTGGTGGCACTTGCTGTTCCCAAGCTCGGCTTGACCTCACGATTTTACGGGTGGTTTCTGTGCCATTATGTCGAATACCTCGATTTGATTGCTAAAGCTCTTCAGCAGCATGTGGTGATCCCTCTTAAGACTTACGTTAGTGATGCTATTGCCCGCATGCAAGCCAGCACCGAGGCCTCCGTATCTACTCGGAAGGAGCTGCCATGGTACCCGTACGACGTCCCGGACTACGCGTAG |
